# Structural and functional conservation of non-lumenized lymphatic endothelial cells in the mammalian leptomeninges

**DOI:** 10.1007/s00401-019-02091-z

**Published:** 2019-11-06

**Authors:** Shannon Shibata-Germanos, James R. Goodman, Alan Grieg, Chintan A. Trivedi, Bridget C. Benson, Sandrine C. Foti, Ana Faro, Raphael F. P. Castellan, Rosa Maria Correra, Melissa Barber, Christiana Ruhrberg, Roy O. Weller, Tammaryn Lashley, Jeffrey J. Iliff, Thomas A. Hawkins, Jason Rihel

**Affiliations:** 1 Department of Cell and Developmental Biology, University College London, London, WC1E 6BT UK; 2 Department of Anaesthesiology and Perioperative Medicine, Oregon Health & Science University, Portland, OR USA; 3 Department of Physiology and Pharmacology, Oregon Health & Science University, Portland, OR USA; 4 The Queen Square Brain Bank for Neurological Disorders, Department of Clinical and Movement Neurosciences, UCL Institute of Neurology, London, UK; 5 Department of Neurodegenerative Disease, UCL Institute of Neurology, London, UK; 6 Knight Cardiovascular Institute, Oregon Health & Science University, Portland, OR USA; 7 UCL Institute of Ophthalmology, University College London, London, UK; 8 Clinical Neurosciences (Neuropathology), Faculty of Medicine, Southampton University Hospitals, Southampton, SO16 6YD UK

**Keywords:** Meningeal lymphatics, Lymphatics, Macrophages, CNS macrophages, CNS lymphatics

## Abstract

The vertebrate CNS is surrounded by the meninges, a protective barrier comprised of the outer dura mater and the inner leptomeninges, which includes the arachnoid and pial layers. While the dura mater contains lymphatic vessels, no conventional lymphatics have been found within the brain or leptomeninges. However, non-lumenized cells called Brain/Mural Lymphatic Endothelial Cells or Fluorescent Granule Perithelial cells (muLECs/BLECs/FGPs) that share a developmental program and gene expression with peripheral lymphatic vessels have been described in the meninges of zebrafish. Here we identify a structurally and functionally similar cell type in the mammalian leptomeninges that we name Leptomeningeal Lymphatic Endothelial Cells (LLEC). As in zebrafish, LLECs express multiple lymphatic markers, containing very large, spherical inclusions, and develop independently from the meningeal macrophage lineage. Mouse LLECs also internalize macromolecules from the cerebrospinal fluid, including Amyloid-β, the toxic driver of Alzheimer’s disease progression. Finally, we identify morphologically similar cells co-expressing LLEC markers in human post-mortem leptomeninges. Given that LLECs share molecular, morphological, and functional characteristics with both lymphatics and macrophages, we propose they represent a novel, evolutionary conserved cell type with potential roles in homeostasis and immune organization of the meninges.

**Electronic supplementary material:**

The online version of this article (10.1007/s00401-019-02091-z) contains supplementary material, which is available to authorized users.

## Introduction

The brain is surrounded by the meninges, a trilaminar compartment comprised of an outer dura mater, a middle arachnoid mater, and an inner layer of pia mater. The meninges not only serve as a protective physical barrier for the central nervous system (CNS) but also participate in the immune surveillance and fluid homeostasis of the cerebrospinal fluid (CSF), which fills the subarachnoid space between the pia and arachnoid layers (collectively known as the leptomeninges). Under steady-state conditions, the meninges are replete with immune cells including macrophages, mast cells, B cells, and T cells that protect the brain from infection and are capable of mounting a rapid and strong neuroinflammatory response. Most CNS immune responses first start within the meninges [51], and meningeal inflammation impacts a variety of diseases, including multiple sclerosis, meningitis, various forms of dementia pathology, stroke, and migraine [7, 14, 44]. Thus, understanding the mechanisms by which the meninges interact with the brain and surrounding CSF during both steady-state and diseased conditions have important clinical and therapeutic implications.

In peripheral tissues, the maintenance of fluid homeostasis is aided by a network of lymphatic vessels that drains fluid and metabolic waste from surrounding tissue and participates in immunity by trafficking leukocytes and T cells [24, 27]. The fibrous outer dura mater of the meninges also contain a lymphatic vascular network lining the venous sinuses [5, 38], and a network of meningeal lymphatic vessels at the base of the skull have recently been described [1, 4, 5]. There is some evidence that dorsal dural lymphatic vessels are capable of draining macromolecules from the CSF [5, 38]. However, other reports fail to detect CSF drainage into dorsal dural lymphatics and suggest alternative drainage routes, such as along blood vessels or olfactory nerves or within lymphatic vessels that pass through the cribriform plate, or into the ventral dural lymphatics at the base of the skull [1, 23, 28, 31, 39, 45]. Dorsal dural lymphatics can also transport immune cells from the meninges and modulate T-cell activation during inflammation, and ablation studies of the dorsal dural lymphatics have demonstrated the importance of these vessels in the promotion of neuro-inflammatory responses in an animal model of multiple sclerosis [37]. Moreover, dorsal dural lymphatic deletion studies suggest they also participate in the drainage of waste from the CSF, including amyloid-β, which forms hallmark extracellular protein aggregates in Alzheimer’s disease [1, 44]. In contrast, enhancing dorsal dural lymphatic drainage of aged mice improved brain clearance and cognitive performance [44], highlighting the untapped therapeutic potential of targeting dural lymphatic function in age and disease.

In contrast to the dura mater, the brain and leptomeninges have never been reported to contain lymphatic vessels. However, a previously undescribed cell type, variously called Brain Lymphatic Endothelial Cells (BLECs), mural LECs (muLECs), or Fluorescent Granule Perithelial Cells (FGPs), that share numerous features of lymphatic endothelium was recently identified in zebrafish [11, 36, 66]. Under normal conditions, BLECs do not lumenize to form vessels but exist as single cells in close association with meningeal blood vessels [11, 36, 66]. However, BLECs do have other molecular, developmental, and functional features of peripheral lymphatic vessels. BLECs express numerous lymphatic endothelial genes, such as the lymphatic markers *prospero homeobox domain protein 1a* (*prox1a*) [26, 69] *vascular endothelial growth factor receptor 3* (*vegfr3*; also known as *fms*-*related tyrosine kinase 4*, or *flt4*) and the *lymphatic vessel endothelial hyaluronan receptor 1* (*lyve1*). Like peripheral lymphatics, BLECs sprout from the venous endothelium and require lymphatic developmental signals such as Collagen and calcium binding EGF domains 1 (Ccbe1) [22], but not the macrophage transcription factor pU.1, to properly form. Functionally, BLECs can internalize macromolecules from the brain in a Mannose Receptor 1a (Mrc1a)-dependent manner [36, 66] and can remove lipids from the blood [11]. They are also important for the proper development of neighbouring meningeal blood vessels [11]. Overall, these data support the view that zebrafish BLECs are a type of lymphatic endothelial cell that reside in the meninges.

Whether BLECs are a fish-specific meningeal cell type or are also present in the leptomeninges of mammals remains an open question. To test whether BLECs are evolutionarily conserved, we examined the expression of BLEC proteins in mouse and human leptomeninges using both confocal and electron microscopy. We discovered cells in both mouse and human that have morphological, ultrastructural, and molecular features of BLECs. As in zebrafish, these cells rapidly internalized macromolecules, including Aβ. To emphasize their evolutionary conservation, lymphatic molecular and functional character, and location within the subarachnoid space, we propose to call this novel mammalian cell type Leptomeningeal Lymphatic Endothelial Cells (LLECs).

## Materials and methods

### Animals

#### Zebrafish

Zebrafish experiments followed guidelines of the animal ethics committees at University College London and were performed in accordance with the UK Animal (Scientific Procedures) Act 1986 under project licences awarded to J.R. and Steve Wilson. The following transgenic and mutant lines were used in this study:

**Table:** 

Transgenic line	References
*Tg(kdr*-*l:HRAS*-*mCherry*-*CAAX)*^*s916*^	[22]
*Tg(flt4*^*BAC*^*:mCitrine)*^*hu7135*^ on *casper* background	[26]
*Tg(mpeg1:Gal4*-*FF)gl25; Tg(UAS*-*E1b:nfsB.mCherry)*^*c264*^ on nacre^−/−^ background	[67]

Embryos, larval and adult zebrafish (Danio rerio) were kept in University College London’s fish facility at 28 °C with a 14 h light and 10 h dark cycle and were fed a diet mix of Safe (bernaqua) Caviar 500–800, Micro Gemma 500, Hikari micro pellets in a mix of 3 to 2 to 1.

#### Mouse

Mouse experiments followed the guidelines of either the animal ethics committees at University College London under project licences awarded to John Parnavelas, Christiana Ruhrberg, Francis Edwards, and Steven Hunt under the UK Animal (Scientific Procedures) Act 1986, or the Institutional Animal Care and Use Committee of Oregon Health & Science University (Jeff Iliff).

The following mouse lines were used in this study:

**Table:** 

Line	References
C57Bl/6	Jackson Laboratories
Tie2-GFP;NG2-DSRed	[25]
*Spi1*^*tm1Ram*^	(PU.1); [43]
*GAD67*-*GFP *+ *ve* (Δ*neo*)	[59]
AKR background	[21]

#### Breeding

For Fig. 3, wild type embryos were obtained from time-mated GAD67-GFP + ve (Δneo) [59] mice, maintained in a C57bl/6J background at Biological Services UCL and GFP-ve embryos were used. For Fig. 4, Spi1^tm1Ram^ (referred to as PU.1^−/−^) [43] transgenic mice and WT controls were maintained on a mixed background (C57Bl6/J;129/Sv). In both experiments, the day of the vaginal plug was considered E0.5.

PU.1 KO mice and wildtype siblings were genotyped as follows:

**Table:** 

Line	OLIGOS	Notes	*T* °C	WT bp	MUT bp	MUT band
Spi1^tm1Ram^	KO2	do wt and mut	60	1237	980	Bottom
Spi1^tm1Ram^	920	pcrs separately		KO2 + 920	KO2 + Neo2	
Spi1^tm1Ram^	Neo2

**Table:** 

PCR	OLIGOS	Sequence (5′-3′)
Spi1^tm1Ram^	KO2	GCCCCGGATGTGCTTCCCTTATCAAAC
Spi1^tm1Ram^	920	TGCCTCGGCCCTGGGAATGTC
Spi1^tm1Ram^	Neo2	CGCACGGGTGTTGGGTCGTTTGTTCGG

### Electron microscopy

#### Zebrafish

Adult zebrafish were killed by terminal anaesthesia followed by cardiac perfusion with either 2% paraformaldehyde/2% EM-grade glutaraldehyde in 0.1 M sodium cacodylate buffer pH7.3 for conventional electron microscopy (EM) or 4% paraformaldehyde (PFA) in 0.1 M phosphate buffer pH7.3 (PB) for immunoEM. For conventional EM, brains were cut into chunks, postfixed in osmium tetroxide and embedded in epoxy (agar100 resin, Agar Scientific) before sectioning and staining with lead citrate. For post-embed immunoEM, sections were embedded in LR-white resin, followed by sectioning, mounting on nickel grids, blocking in normal goat serum (NGS) and incubation with polyclonal rabbit anti-GFP primary antibody (TP401; Amsbio) followed by anti-rabbit 20 nm gold-labelled secondary (TAAB GBL024/1).

#### Mouse

Founder C57BL/6j mice were purchased from Charles River and male offspring were bred and maintained at UCL on a 12-h light/12-h dark cycle with ad libitum supply of food (Envigo 2018 Teklad global 18% protein rodent diet) and water. Mice were deeply anaesthetised with Euthatal then transcardially perfused with heparinised (5000 U/l) physiological saline, followed by 4% PFA in PB pH 7.3 prepared fresh on day of perfusion. Following this, brains were dissected and postfixed by immersion in the same fixative mix overnight at 4 °C. Immunohistochemistry was carried out in PB on floating 100 µm coronal vibratome sections using rabbit anti-LYVE1 antibody (11-034, Angiobio). Nanogold (1.4 nm; Nanoprobes) goat anti-rabbit secondary was used and detected using HQ silver enhancement kit (Nanoprobes) following manufacturer’s instructions before postfixation using 1% EM-grade glutaraldehyde and conventional epoxy-based EM processing and sectioning. Ultrathin sections were stained lightly for contrast using lead citrate.

#### EM imaging

Both zebrafish and mouse sections were examined using JEOL1010 microscope with a Gatan Orius camera. The 32 bit digital image files (*.dm3) were processed using Fiji-ImageJ with minimal contrast adjustments and scale bar additions. Pseudo colouring, thresholding (to highlight gold/silver labelling), and other labelling was carried out using Adobe Photoshop.

### Auto-fluorescence inclusion check

#### Zebrafish

5-month-old WT AB zebrafish fed a diet mix of Safe (bernaqua) Caviar 500–800, Micro Gemma 500, Hikari micro pellets in a mix of 3-to-2-to-1, were terminally anaesthetized in MS222 (Sigma, #A5040) and brain dissected in ice cold 0.1% phosphate buffered saline (PBS). Dissected brains were first examined in a petri dish on an epifluorescence microscope, and then placed in a 4% paraformaldehyde/4% sucrose solution O/N at 4 °C. Brains were then washed 3 × in PBS before mounting in 2% low-melting agarose (ThermoFischer, #16520100) dissolved in PBS within glass rings that were then cover-slipped.

#### Mouse

Founder C57BL/6j mice were purchased from Charles River and male offsprings were bred and maintained at UCL on a 12-h light/12-h dark cycle with ad libitum supply of food (Envigo 2018 Teklad global 18% protein rodent diet) and water. Mice were deeply anaesthetised with Euthatal then transcardially perfused with heparinised (5000 U/l) physiological saline, followed by 4% PFA in PBS, prepared fresh on day of perfusion. Freshly dissected brains of 5-month-old mice were immersed in 4% PFA/PBS, incubated for 2 h at 4 °C on ice, washed twice with PBS and then immersed in 30% sucrose/PBS and kept at 4 °C. Brains were then washed 3× in PBS before mounting in 2% low-melting agarose (ThermoFischer, #16520100) dissolved in PBS within glass rings that were then cover-slipped.

#### Auto-fluorescence imaging

Images were obtained from an Olympus MVX10 with a standard GFP bandpass at 6× and after fixation with a Leica DM LB epi-fluorescence microscope with a DFC310 FX camera and standard GFP ex/em bandpass filter cube with 10×–60× air lenses. A Leica SP8 LSCM was then utilised with a 10× dry lens and 25× water immersion lens, applying a 500–588 nm bandpass and exciting with a 488 nm argon laser and zoom of 0.75 and 3.

### Fluorescent in situ hybridization

Founder C57BL/6j mice were purchased from Charles River and male offspring were bred and maintained at UCL on a 12-h light/12-h dark cycle with ad libitum supply of food (Envigo 2018 Teklad global 18% protein rodent diet) and water. Mice were deeply anaesthetised with Euthatal then transcardially perfused with heparinised (5000 U/l) physiological saline, followed by 10% formalin solution (4% PFA in PBS, prepared fresh on day of perfusion). Freshly dissected brain hemispheres of 5 month-old mice were immersed in 4% PFA/PBS (all PBS was DEPC treated) solution, incubated for 24 h at 4 °C, washed twice with PBS and then immersed in 30% sucrose/PBS for 1–2 days at 4 °C. Fixed brain tissue was then washed twice in PBS, frozen in pre-cooled isopentane (2-methylbutane, Sigma-Aldrich) on dry ice and stored at − 80 °C. Frozen brain hemispheres were embedded in OCT and 30 μm coronal sections were cut on a Leica CM1850 cryostat.

Digoxigenin and fluorescein-labelled probes were made via standard protocols, amplified by PCR from cDNA using primer pairs found on Allen Mouse Brain Atlas [34] as described below.

The in situ hybridization protocol was adapted from a previous published protocol [3] with the following modifications:

**Table:** 

Gene name	Forward pair	Reverse primer (with T3 sequence)	Probe length
*Vegfr3*	5′CAAGACCTGCTTTCTCTGACCT-3′	5′AATTAACCCTCACTAAAGGGTCCTGGATATGGAGGCTGTAGT-3′	763 bp
*Prox1*	5′TATATATTTGTGTGGGGGAGGC-3′	5′AATTAACCCTCACTAAAGGGGCAACTAGTGACAAAGCACAGG-3′	647 bp

30 µm-thick sections were transferred to a 12-well falcon plate into PBS/Tween 20 (PBST) and washed 3 × 10 min to remove sucrose. Brain sections were pre-hybridised overnight in hybridization solution at 60 °C, in a hybridization oven gently rocking until the sections sank. The following day riboprobes were pre-heated in blocks to 80 °C for 10 min prior to being mixed in hybridization buffer, at a final concentration of 100–300 ng/µL.

Brain sections were hybridised overnight at 60 °C in a hybridization oven with gentle rocking. Probe removal was done with a series of hot washes in preheated wash solution (50% Formamide, 1 × SSC, 0.1%Tween-20) at 65 °C for 3 × 1 h. Sections were subsequently washed in Maleic Acid Buffer (100 mM Maleic Acid, pH 7.5) and blocked for at least 1 h at RT in blocking solution (2% Boehringer Blocking Reagent in Maleic Acid Buffer; 11096176001, Roche). Anti-Fluorescein-POD antibody (11426346910, Sigma) was applied at 1:500 dilution in blocking solution, and incubated overnight at 4 °C. Unbound antibody was subsequently removed in a series of PBST washes at RT. Detection of fluorescein-labelled riboprobes was done using as substrates the bench-made fluorescein tyramide (green) in some wells and Cy3 tyramide (red) in others, as previously described [33]. Brain sections were incubated for 20 min in either fluorescein tyramide (1:500) or cy3 (1:100) diluted in PBST, at RT and with gentle shaking, protect from light. 2 µL of 0.5% H_2_O_2_ in PBST was added to 1 mL of the tyramide solutions for a final concentration of 0.001%. The tyramide reaction ran for 40 min protected from light and without agitation. Samples were subsequently rinsed and washed 3× 15 min in PBST. Samples were then washed for 10 min each in 30%, 50%, 75% and 100% methanol in PBST.

Sections were then incubated in a solution of 1% H_2_O_2_ in methanol for 30 min to inactivate the first peroxidase. Samples were then rehydrated in the wells in a reverse order for 10 min each at RT then washed 2× in PBST for the removal of all traces of methanol. Brain slices were incubated in blocking buffer for 1 h at RT. Anti-DIG POD (11207733910, Sigma) was added into the appropriate wells with the block at 1:1000 O/N at 4 °C. The following morning samples were washed 4 × 30 min in PBST at RT. Samples were washed several times in PBST and then mounted onto glass slides with coverslips using an anti-fade mounting media (CitiFluor, UK) and kept at 4 °C in the dark until imaging.

Brains from single and double fluorescent ISH were imaged on a Leica SP8 LSCM with 25× water of 63× oil immersion objectives.

### Aβ injections and infusions

#### Zebrafish Aβ injection

Aβ injections were carried out with a Pneumatic PicoSpritzer III (Parker Instrumentation) with glass capillary needles (Harvard Apparatus # GC100F-15) prepared with a Micropipette Puller (Sutter Instruments, # P-87) HiLyte647-conjugated Aβ1-40 (Anaspec) was constituted in PBS, diluted to 1 mg/mL and kept on ice until the point of injection. The injected embryos were anaesthetized in 2% low-melting agarose (ThermoFischer, #16520100) dissolved in embryo medium containing 42 mg/L MS222 (Sigma, #A5040) and injected with a total volume of 1 nL per injected bolus. Needles were inserted into the brain in a sloped angle into the CSF directly dorsal lateral to the posterior region of the hindbrain ventricle.

Live larval fish and whole mounts were imaged with a Leica SP8 laser scanning confocal microscope (LSCM) using 25× and 40× water immersion objectives.

#### Mouse intracisternal tracer infusion

For experiments involving mice, 2–6 month old male and female C57Bl/6 or NG2-DsRed mice from Jackson Laboratories were used after several days of acclimation to their home environment.

Anaesthesia was induced with 3% isoflurane and after a surgical plane was achieved, isoflurane was reduced to 1.5%. Mice were placed in a 3-point stereotaxic apparatus and fur on the posterior head and cervical regions was removed with a chemical depilatory agent. Mice were placed under a heating lamp and core body temperature was monitored with a digital rectal thermometer throughout the experiment.

For intracisternal infusions, a 2-cm midline incision was made along the posterior head and neck. Skin was retracted laterally and the levator auris longus, platysma, acromiotrapezius, and splenius muscles were sectioned and reflected laterally with 6-0 braided suture to expose the atlanto-occipital membrane. The mouse’s nose was lowered approximately 20° and posterior longitudinal ligament was dissected to optimize visualization of the cisterna magna. A 30 ½ ga. needle was inserted into 12 inches of PE-10 tubing connected to a 10 µL gas chromatography syringe (Hamilton). A solution of Aβ_1-40_ HiLyte-555 (Anaspec) or10 kDa Cascade blue dextran (Thermofischer D1976) was backfilled into the tubing and the bevel of the needle was inserted into the cisterna magna. Supplementary Fig. 5 was from a 1:1 co-infused solution. Placement was confirmed and 2 μL of solution was infused into the cisterna magna at a rate of 1 μL per minute. Mice were sacrificed 1 hour after initiation of infusion by intracardiac infusion of normal saline and 4% paraformaldehyde for fixation. After dissection of cranium, the brain tissue was gently removed, fixed in 4% PFA overnight, and prepped for immunohistochemistry.

### Immunohistochemistry

#### Zebrafish

Coronal cryosections of adult brains from 14 month old *Tg(flt4:mCitrine); Tg(kdr*-*l:mCherry)* fish were labelled as per [36]. Primary antibodies used were α-RFP (1:2000, Rockland USA, #600-901-379), α-GFP (1:2000, Nacalai Tesque, 04404–84). Secondary antibodies used were α-chicken Alexa Fluor 568 (1:2000, ThermoFischer, #A11041), α-rat Alexa Fluor 488 (1:2000, ThermoFischer, A11006). DAPI was used at 1:1000 dilution (ThermoFischer, D1306).

Zebrafish brain sections were imaged with a Zeiss Airyscan 880 LSCM using a 63× oil immersion objective and a Leica SP8 LSCM using 25× water of 63× oil immersion objectives.

#### Mouse

##### Antibodies (Figs. 1, 3, 4, 6, and Supplemental Figs. 2, 3)

**Fig. 1:**
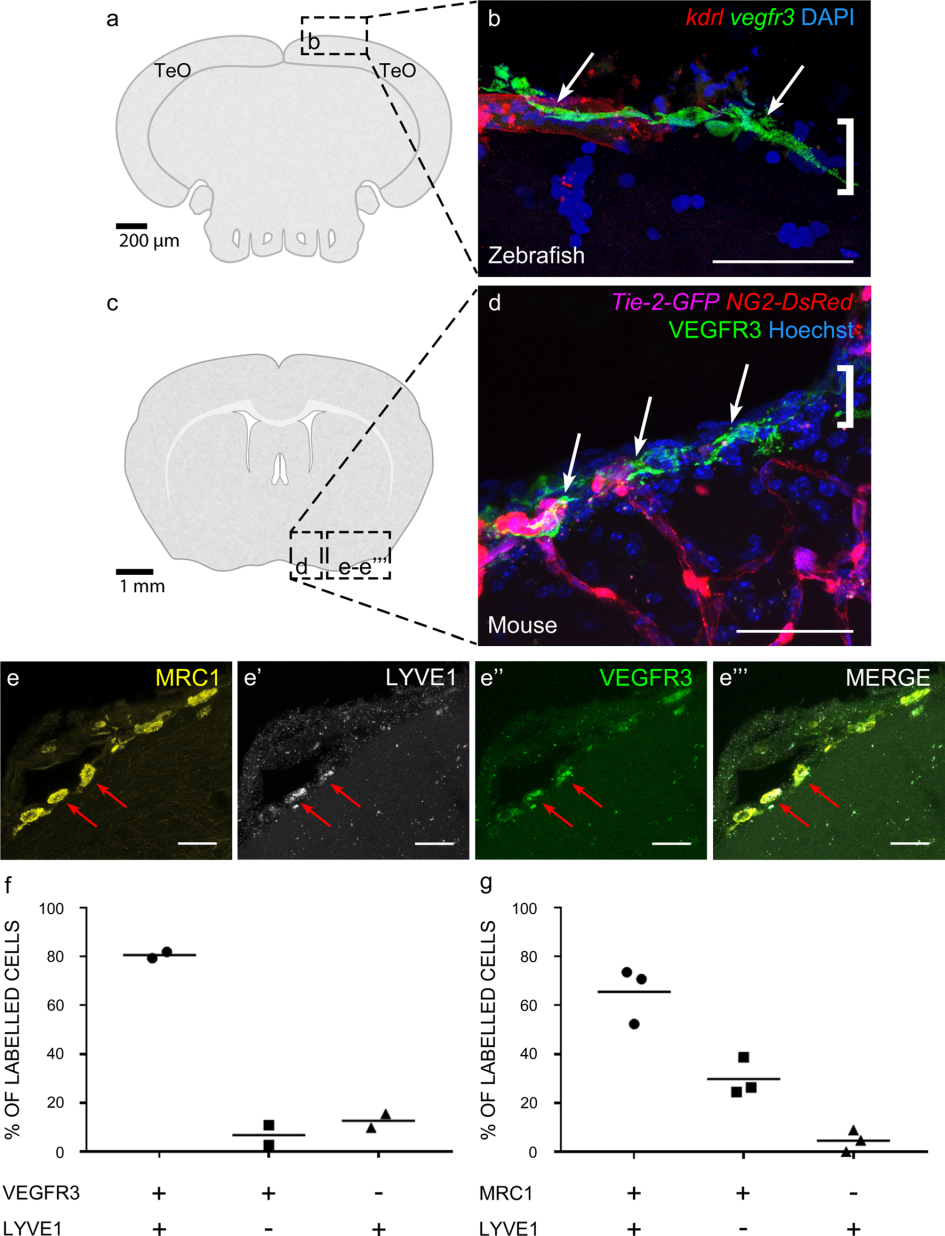
Cells with BLEC molecular markers are present within the mouse leptomeninges. **a** Coronal brain section of adult zebrafish brain indicating the imaging area in the dorsal optic tectum (TeO). **b** A 14 month old *Tg(kdr*-*l:mCherry); Tg(flt4:mCitrine)* double transgenic zebrafish has cells in the meninges (white bracket) that express *flt4*/*vegfr3* (α-GFP, green) near *kdr*-*l* positive (α-RFP, red) blood vessels. DAPI (blue) labels the nuclei. Scale = 50 µm. **c** Coronal mouse brain section showing the imaging areas of the meninges. **d** As revealed by IHC, 17-week-old mouse brains express VEGFR3 (green) in the meninges (white bracket). Tie2-GFP;NG2-DsRed double reporter mice were used to distinguish arteries and veins. NG2 (red) labels pericytes and smooth muscle cells, Tie2 (magenta) labels vascular endothelial cells, and Hoechst (blue) stains nuclei. The image is rotated with the parenchyma at the bottom for ease of comparison with panel b. Scale = 50 µm. **e-e′′′** As revealed by IHC, cells of the meninges co-express MRC1 (**e**, yellow), LYVE1 (**e′**, white), and VEGFR3 (**e′′**, green). Red arrows highlight cells expressing these three markers. The images are rotated with the parenchyma at the bottom. scale = 30 µm. **f**, **g** Quantification of the relative numbers of single and double-labelled cells in 2-month old mouse meninges. VEGFR3 and LYVE1 cell counts were from *n* = 2 brains, 3 coronal sections (10 area images)/brain. MRC1 and LYVE1 cell counts were from *n* = 3 brains, 3 coronal sections (4 area images)/brain. The mean values for each set are depicted

The primary antibodies used in most mouse IHC experiments are as follows: MR1 (1:100–200, Abcam ab64693), VEGFR3 (1:100–200, R&D Systems, AF743), PROX1 (1:200, Abcam ab38692): LYVE1(1:200–1:1000 Thermofischer 14-0443-80 ALY7), MR1 (1:500 RandD AF2535). MRC1 (R&D Systems AF2535RD), PROX1 (1:500-1000 Novus NBP1-18605), LAMININ (1:200 Chemicon AB19012MI), LYVE1 (1:200 Novus NB110-61026) PODOPLANIN (1:500, eBioscience 8.1.1 14-5381-82). The following fluorescent secondaries were applied at dilutions of 1:500: Donkey α-Goat Alexa Fluor 633 (Invitrogen A21082), Donkey α-Rabbit Alexa Fluor 488 (Invitrogen A21206), Donkey α-Rat DyLight 550 (Invitrogen SA5-10027) and Goat α-Syrian Hamster FITC, (eBioscience 11-4211-85). DAPI (1:1000; Life Technologies, D3571) or Hoechst 33342 at 1:5000 (Fig. 1)was applied for nuclear labelling.

##### For adult cryosections of WT and Aβ infused brains (Figs. 1, 6)

30 µm brain cryosections were washed in phosphate buffered saline (pH 7.4) and blocked in blocking buffer (0.3% Triton X-100 PBS + 5% donkey serum and 2.5% bovine serum albumin) overnight at 4 °C. Sections were incubated in primary antibodies overnight at 4 °C. After washing slides in PBS (pH 7.4), sections were incubated in secondary antibody for 4 h at room temperature. Sections were washed in PBS (pH 7.4), stained with Hoecht 33342 (1:5000) for 10 min and mounted in mowiol 4-88 (Sigma).

The sections were imaged with a Zeiss Airyscan 880 LSCM using a 63× oil immersion objective or Leica SP8 LSCM using 25× water and 63× oil immersion objectives.

##### IHC on embryonic mouse samples (Figs. 3, 4 and Supplementary Fig. 4)

Pregnant mothers at 12.5, 13.5, and 18 days of gestation were euthanized by cervical dislocation, and embryos were immediately removed from the uterus and transferred to 1× 0.1% phosphate buffered saline on ice. Whole embryonic heads were fixed in 4% paraformaldehyde, made up in PB for 2 h on ice, rinsed 3×15 min in 1× PBS at RT and then cryoprotected in 30% sucrose in PBS overnight at 4 °C on a shaker. Whole heads were embedded and frozen in either OCT (Tissue Tek; Fig. 3) or pre-cooled isopentane (2-methylbutane, Sigma-Aldrich; Fig. 4) on dry ice and stored at − 80 °C. 30 μm coronal sections were cut on a Leica CM1850 cryostat onto glass slides, allowed to dry for an hour at RT and then stored at − 80 °C.

Slides with sections were thawed to RT for 20 min and rehydrated via 3× PB washes which were applied to sections to remove excess sucrose. Sections were then blocked in 1 mL of block solution consisting of 3% donkey serum (D9663-10ML Sigma), 0.3% Triton X-100 in PB for an hour. Primary antibodies were added into 1 mL tris buffered saline/Tween 20 (TBST: comprised of 50 mM Tris, 50 mM NaCl, 0.1% Tween 20) overnight at room temperature. The primary antibody solution was removed, the sample was washed 3× 10 min in PB, and secondary antibody solution (all at 1:200 concentration) TBST was added for 2 h in the dark at room temperature. Secondary solution was then removed and washed 3× 10 min in PB. Sections were then mounted onto gelatin-coated slides in PB, removing excess liquid and subsequently cover-slipped with mounting media (Citifluor, UK) and stored at 4 °C until imaging with a Zeiss Airyscan 880 LSCM using a 63× oil immersion objective and a Leica SP8 LSCM using 25× water and 63× oil immersion objectives.

##### IHC on mouse paraffin-embedded sections (Supplemental Fig. 1a, c)

6 month-old mouse brains in an AKR background were sectioned and paraffin-embedded [21]. Sections on slides were deparaffinised in xylene and rehydrated using graded alcohols. Immunohistochemistry for all antibodies required pre-treatment with a pressure cooker for 10 min in citrate buffer pH 6.0. Endogenous peroxidase activity was blocked in 0.3% H_2_O_2_ in methanol for 10 min and non-specific binding with 10% dried milk solution. For this specific experiment, tissue sections were incubated with primary antibodies against human proteins that cross-react with mouse; PROX-1 (1:400; Acris), or VEGFR3 (1:40; R&D Systems) for 1 h at RT, followed by biotinylated anti-rabbit IgG (1:200; Dako) or biotinylated anti-mouse IgG (1:200; Dako) for 30 min at RT and Avidin–Biotin complex (30 min; Dako). Colour was developed with di-aminobenzidine/H_2_O_2_ [32].

Images were acquired using a Nikon Eclipse Ni microscope using 10×, 20×, and 40× air objectives and 60× oil objective.

### Human brain immunohistochemistry

#### Cases

Brain tissue from four normal controls was obtained through the brain donation program at Queen Square Brain Bank for Neurological Disorders (QSBB), Department of Clinical and Movement Neuroscience, UCL Queen Square Institute of Neurology. The demographic data for all cases are shown in Table 1. Ethical approval for the study was obtained from the Local Research Ethics Committee of the National Hospital for Neurology and Neurosurgery.

**Table 1:** Human case data

Case	QSBB number	Gender	Age at death	PM delay (hrs:mins)	Brain weight (g)	Thal phase	Braak and Braak stage	CERAD	ABC score
1	P80-17	M	88	27:30	1330	3	4	2	A2B2C2
2	P24-18	F	92	32:30	1450	3	2	1	A2B1C1
3	P33-18	M	89	47:05	1356	3	2	1	A2B1C1
4	P53/06	M	34	14:00	n/a	0	0	0	A0B0C0
5	P17/07	M	38	80:35	1581	0	0	0	A0B0C0
6	P78/06	F	68	45:05	1330	0	0	0	A0B0C0

#### Sample preparation and IHC

Formalin-fixed, paraffin-embedded tissue Sections (8 µm) from the frontal and occipital cortices were cut from cases 1–6 listed in Table 1. Sections were deparaffinised in xylene and rehydrated using graded alcohols. Immunohistochemistry for all antibodies required pre-treatment with a pressure cooker for 10 min in citrate buffer pH 6.0. Endogenous peroxidase activity was blocked in 0.3% H_2_O_2_ in methanol for 10 min and non-specific binding with 10% dried milk solution. Tissue sections were incubated with primary antibodies; MRC1 (1:1000; R&D systems), PROX1 (1:400; Acris), LYVE1 (1:50; Abcam), LYVE1(1:200; R&D Systems), PDPN (1:350; Sigma-Aldrich), and VEGFR3 (1:40; R&D Systems) for 1 h at RT, followed by biotinylated anti-rabbit IgG (1:200; Dako) or biotinylated anti-mouse IgG (1:200; Dako) for 30 min at RT and Avidin–Biotin complex (30 min; Dako). Colour was developed with di-aminobenzidine/H_2_O_2_ [32].

Images were acquired using a Nikon Eclipse Ni microscope using 10×, 20×, and 40× air objectives and 60× oil objective.

#### Triple immunofluorescence

Formalin-fixed paraffin-embedded tissue was sectioned at 8 µm and 20 µm. Sections were deparaffinized in xylene and rehydrated using a gradient of alcohol. Endogenous peroxidases were first quenched with 0.3% H_2_O_2_ in methanol for 10 min. Antigen retrieval was undertaken in a pressure cooker for 10 min at maximum pressure in 10 mM citrate buffer (pH 6.0) and sections were blocked in 10% non-fat milk for 30 min. Due to differing species combinations the following combinations of primary antibodies were used to assess colocalization; LYVE1 (Rabbit)/MRC1 (Mouse)/VEGFR3 (Goat); LYVE1(Goat)/MRC1(Mouse)/PROX1(Rabbit); VEGFR3 (Goat)/PROX1(Rabbit)/MRC1(Mouse). After washing, sections were sequentially incubated in AlexaFluor goat anti-rabbit 635, AlexaFluor goat anti-mouse 568 and AlexaFluor donkey anti-goat 488 at 1:1000 for 1 h at room temperature.

### Image processing and statistical analysis

Post-image processing using raw data, including image thresholding and maximum intensity *z*-stack projections (adjusted for printing), were performed using ImageJ 7.7.2 software. Iso surface rendered images were processed via Imaris 8.4.1 (Bitplane A.G.). Images and figures were assembled using Adobe Photoshop and Adobe Illustrator. Statistical analysis and graphs were prepared using Microsoft Excel, GraphPad PRISM version 7.05 and the R bootstrap library using Q3.R. Bootstrapping was run without an assumption of normality, with 100 k iterations and *α* = 0.05.

## Results

### BLECs are present within the mouse leptomeninges

In zebrafish, BLECs are unambiguously identified as *vegfr3* expressing cells that are in close association with meningeal blood vessels (Fig. 1a, b). Immunohistochemistry (IHC) on the cortical leptomeninges from a 17-week-old Tie2-GFP;NG2-DsRed double reporter mouse [25] using antibodies against mouse VEGFR3 also labelled cells that resided in close proximity to Tyrosine Protein Kinase Receptor 2 (Tie-2)-positive blood vessels (Fig. 1c, d). As in zebrafish, these cells did not associate with vessels that had penetrated into the brain. These cells did not correspond to Neural/Glial Antigen 2 (NG2)-positive pericytes or smooth muscle cells. Similar results were obtained with alternative VEGFR3 antibodies on paraffin-embedded tissue (Supplementary Fig. 1a, b) as well as by in situ hybridization against *Vegfr3* mRNA (Supplementary Fig. 1d, e), ruling out antibody staining artefacts. To confirm the identity of these VEGFR3-positive cells as the mammalian BLEC homologue, we also examined mouse leptomeninges for the co-expression of VEGFR3, MRC1, and LYVE1, which are BLEC-associated markers in zebrafish. Although leptomeningeal cells expressed a heterogeneous combination of markers, numerous cells co-expressed all three tested BLEC markers (Fig. 1e–e’’’). Cell counts from independent brains found that VEGFR3 co-localized with LYVE1 95% (93–97%, *n* = 2 brains) of the time, while MRC1 co-localized with LYVE1 72% (70–77%, *n* = 3) of the time (Fig. 1f, g). Because MRC1 and LYVE1 are expressed in subsets of macrophages and dendritic cells, and VEGFR3 has been reported in peripheral, but not meningeal, macrophages [30, 71] we examined the leptomeninges for expression of the additional lymphatic endothelial marker, PROX1, which along with VEGFR3 has not been described in CNS border macrophages [18, 29]. IHC on paraffin-embedded sections with antibodies against PROX1 labelled leptomeningeal cells (Supplementary Fig. 1c). Double in situ hybridization confirmed that these *Prox1*-positive cells also expressed *Vegfr3* mRNA (Supplementary Fig. 1f, h). Finally, we tried antibodies against the widely-used LEC marker, PODOPLANIN (PDPN), but, similar to a previous report [61], found in mouse tissue a nearly ubiquitous expression in the pia that extended into the glia limitans (Supplementary Fig. 2). Thus, the use of PDPN to identify individual cells in the meninges was not possible.

These data demonstrate that mouse leptomeninges contain a cell type that co-expresses at least three and likely four zebrafish BLEC markers that have not been described as co-expressed in other known leptomeningeal cell types. However, Mato/Fluorescent Granule Perithelial (FGP) cells, a phagocytic cell type of the mammalian meninges with auto-fluorescent inclusions, have been proposed to be the mammalian equivalent of BLECs [66]. We therefore tested whether zebrafish and mammalian BLECs auto-fluoresced under an epifluorescence microscope as was applied by Mato and colleagues [42] as well as by Venero Galanternik and colleagues [66]. No auto-fluorescent inclusions were detected with standard GFP, RFP and UV band passes. We also applied a 500–588 nm bandpass with high-intensity laser exposure under confocal microscopy. We detected auto-fluorescence from the blood vasculature but failed to observe auto-fluorescent inclusions in the meninges of either zebrafish (Supplementary Fig. 3) or mouse (Supplementary Video 1), suggesting that auto-fluorescence is not a consistent feature of this cell type.

Based on their expression profile similarity to zebrafish BLECs, we named these mammalian cells Leptomeningeal Lymphatic Endothelial Cells (LLECs) and sought to further characterize whether this cell type shared other features of BLECs.

### Ultrastructural properties of BLEC/LLECs are conserved in mice

**Fig. 2:**
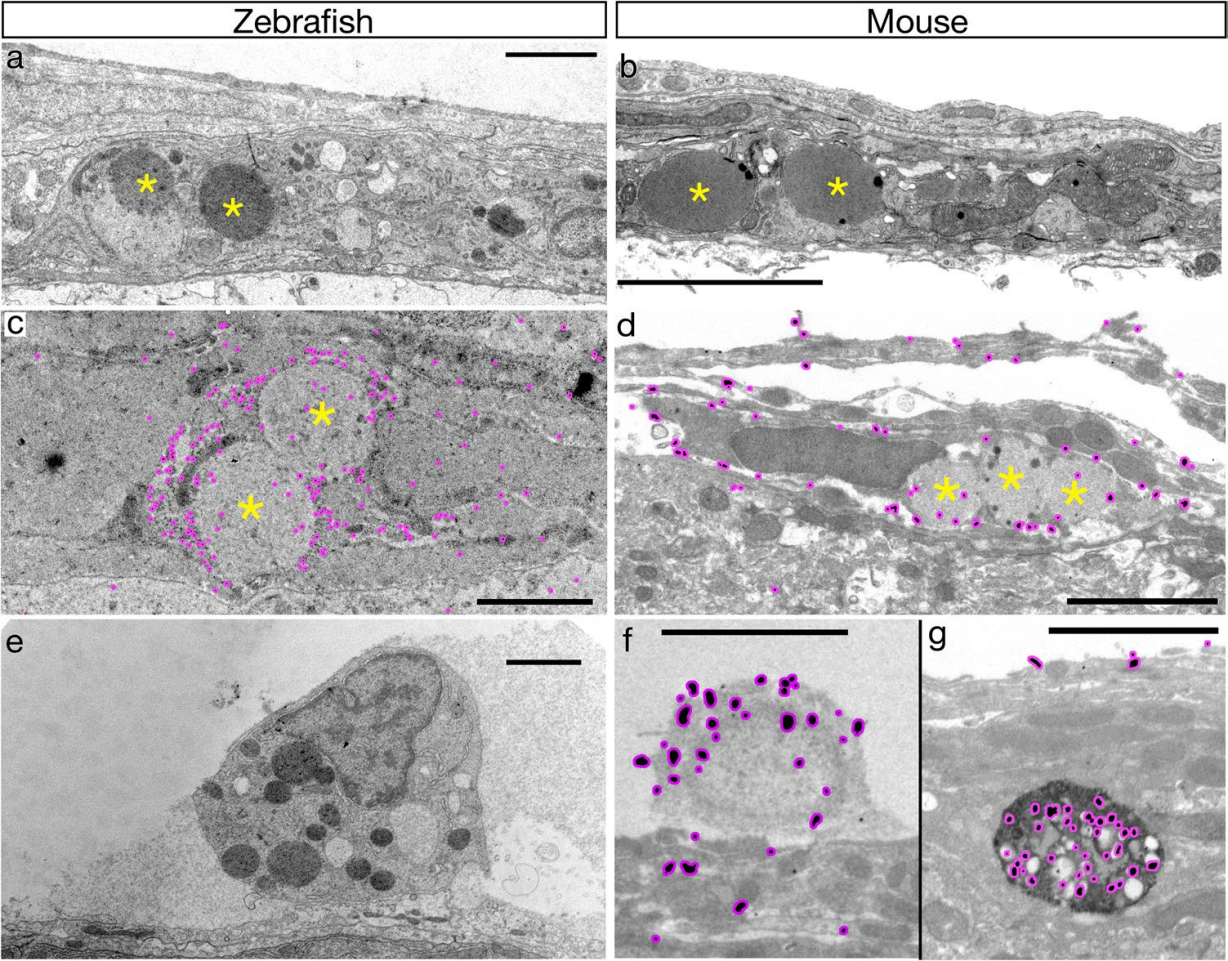
Ultrastructural properties of BLECs/LLECs are conserved in zebrafish and mice. **a** Representative TEM micrograph of adult zebrafish meninges. The large, round, densely and homogenously-stained inclusions (yellow asterisks) of a putative BLEC are indicated. Scale bar = 2.5 µm (in all images). *n* = 2 brains. **b** Representative TEM micrograph of adult mouse leptomeninges. Putative LLECs have their large inclusions marked with yellow asterisks. *n* = 2 brains. **c** Cells with large inclusions (yellow asterisks) characteristic of BLECs are immunologically labelled with post-embed detection of GFP in Tg(*flt4:gfp*) adult brains using a 20 nm gold-labelled secondary. Minimal contrast staining was used to prevent obscuring of the nanogold particles (highlighted in magenta). *n* = 2 brains. **d** Mouse meningeal cells with large inclusions (yellow asterisks) are labelled by pre-embed detection of LYVE1 using a nanogold secondary with silver-enhancement. The silver grains are highlighted with magenta. Note the LYVE1-positive cell has silver grains present near the outer membrane. *n* = 2 brains. **e** An example of a GFP-negative cell in zebrafish Tg(*flt4:gfp*) adult meninges with similar ultrastructural properties to macrophages. *n* = 2 brains. **f** A second population of LYVE1-positive cells in the mouse leptomeninges contain multiple small, irregular, heterogeneous vesicles with silver grains (magenta) present within the cytoplasm and vesicles. These cells are ultrastructurally similar to macrophages (**e**). *n* = 2 brains

As previously described [11, 36], transmission electron microscopy (TEM) of zebrafish meninges identified cells outside of blood vessel basal laminae that possessed distinctively large, homogenous, electron-dense spherical inclusions (Fig. 2a). TEM of adult mouse cortical leptomeninges also identified cells with large, homogenous spherical inclusions (Fig. 2b). Although we cannot rule out their existence, we have not been able to identify other cell types containing such large, homogenous inclusions. To confirm unambiguously that these cells corresponded to BLECs, we first performed immunogold EM for YFP on the meninges of zebrafish carrying a transgenic BLEC reporter [*Tg (flt4:mCitrine)*] and found the cells with large, round inclusions were labelled (Fig. 2c, asterisks). In mouse leptomeninges, immunogold EM with antibodies against LYVE1 labelled cells containing large, homogeneous inclusions that are features of BLECs (Fig. 2d, asterisks). LYVE1 is also expressed in meningeal macrophages, and immunogold EM additionally labelled an ultrastructurally distinct population of rounded cells that possess numerous, irregularly shaped, heterogenous inclusions (Fig. 2f, g) that are characteristic of macrophages. Consistent with this interpretation, a similar macrophage-like cell type was observed in the meninges of Tg(*flt4:mcitrine*) zebrafish, but these cells were never labelled by immuno-EM against YFP, indicating that these cells lack canonical BLEC marker expression. Together, these results show that the ultrastructural distinctiveness of zebrafish BLECs is conserved in mouse LLECs and that these cells are a distinct population from CNS macrophages in both species.

### Development of murine LLECs is distinct from cells of myeloid origin

To further explore whether mouse LLECs are distinct from border macrophages, we investigated the developmental origins of these cells. In zebrafish and mice, meningeal macrophages are derived from a monocytic lineage and therefore require the transcription factor PU.1 to differentiate [2, 18, 43]. In contrast, zebrafish BLEC development does not require pU.1, consistent with their distinct ontological origin that is more similar to peripheral lymphatic vessels [36, 66]. We therefore examined whether cells that express LLEC markers were still present in the meninges of PU.1 knockout mice, which lack all macrophages [43].

**Fig. 3:**
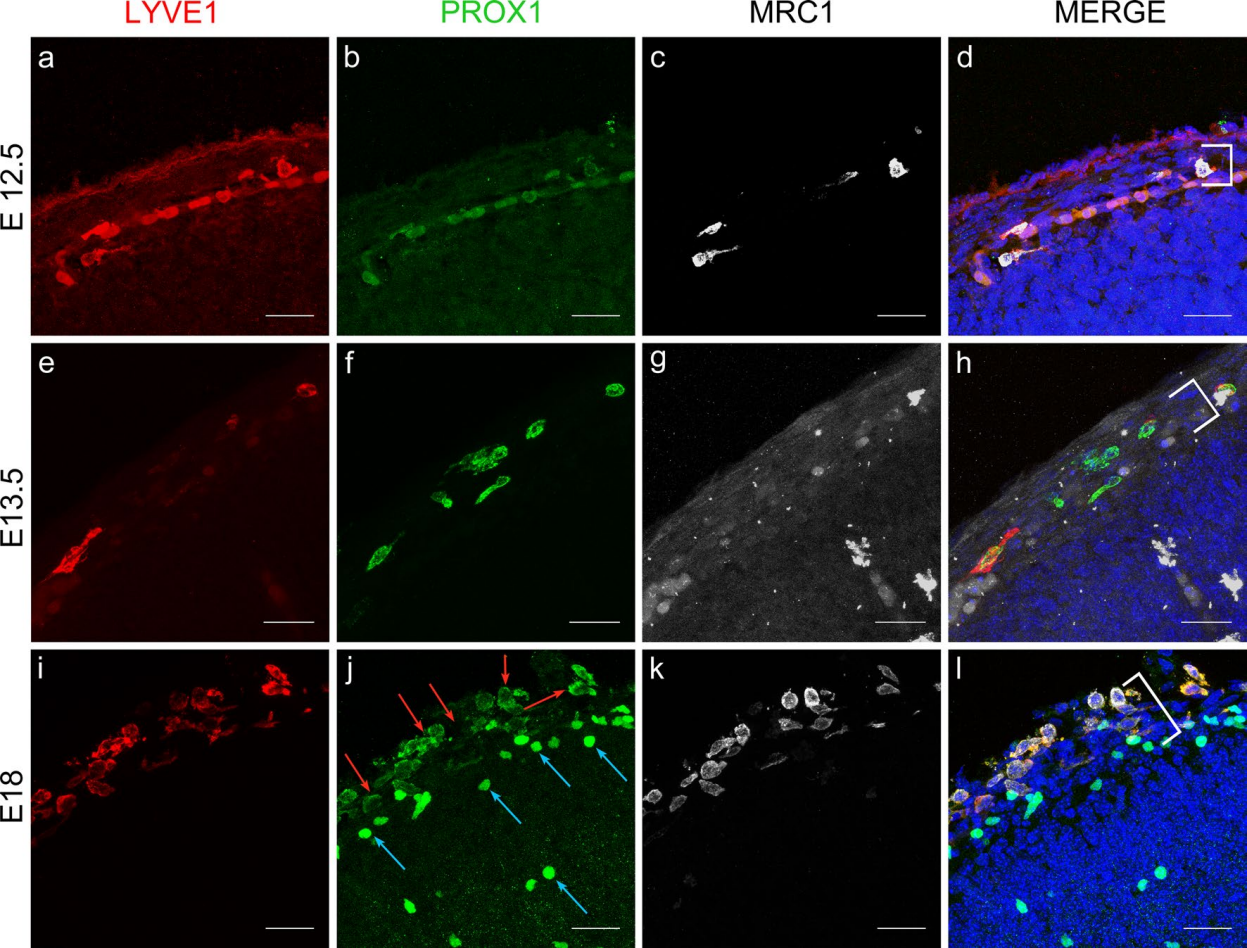
Cells expressing LLEC markers are present within embryonic mouse leptomeninges. **a**–**d** As revealed by IHC, E12.5 mouse meninges (white bracket) contain cells that co-express LYVE1 (red), PROX1 (green), and MRC1 (white). DAPI marks the nuclei in blue (**d**). Scale = 30 µm. *n* = 3 brains. **e**–**h** E13.5 mouse meninges (white bracket) contain cells that co-express LYVE1 (red), PROX1 (green), and MRC1 (white). DAPI marks the nuclei in blue (**h**). Scale = 30 µm. *n* = 3 brains. **i**–**l** E18 mouse meninges (white bracket) contain cells that co-express LYVE1 (red), PROX1 (green), and MRC1 (white). PROX1 is cytoplasmic (**j**, red arrows) in the meninges but nuclear in neurons (**j**, blue arrows). DAPI marks the nuclei in blue (**l**). Scale = 30 µm. *n* = 3 brains

**Fig. 4:**
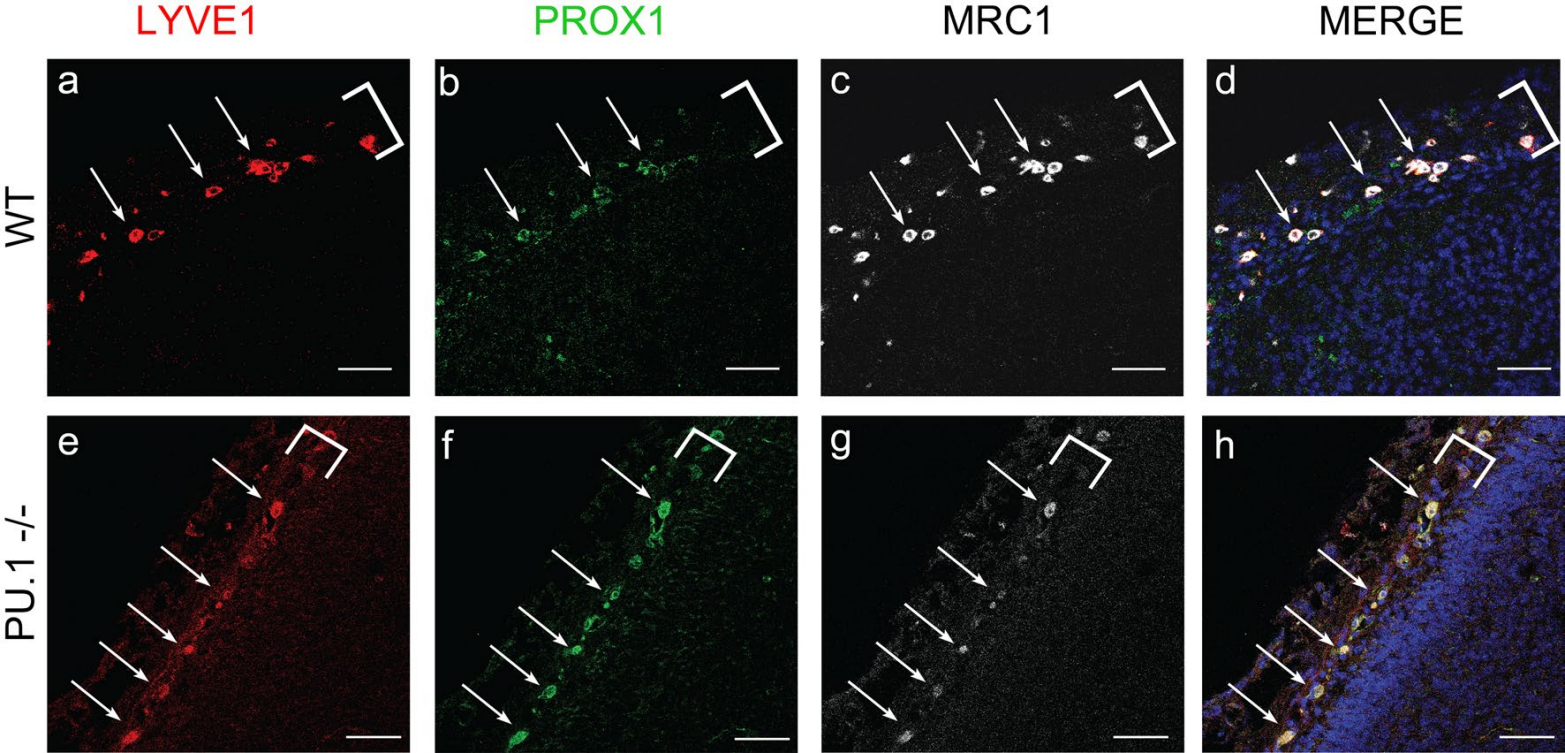
Mouse LLECs develop independent of the transcription factor PU.1. **a**–**d** As detected by IHC, E15.5 wild-type mice have cells that co-express LYVE1 (**a**, red) PROX1 (**b**, green) and MRC1 (**c**, white) in the developing meninges (white bracket). DAPI labels nuclei blue in the merged image (**d**). White arrows indicate cells with these three markers. Scale = 50 µm. *n* = 2 brains. **e–h**) The meninges (white bracket) of E15.5 PU.1 knockout siblings contain many cells (white arrows) that co-express LYVE1 (**e**, red), PROX1 (**f**, green), and MRC1 (**g**, white). Scale = 50 µm. *n* = 3 brains

Because PU.1 knockout mice are perinatal lethal, we first confirmed the presence of LLECs in the developing meninges (E12.5–E18.5) using IHC against LYVE1, PROX1, and MRC1 and found cells expressing these three markers as early as E12.5 (Fig. 3a–l). Similarly, in PU.1 knockout mice, which lack macrophages and other differentiated myeloid cells [18, 43], cells were still detected that co-expressed at least three BLEC markers (Fig. 4e–h), although MRC1 expression appeared reduced compared to wild-type controls (Fig. 4c, g). We confirmed these PU.1-independent cells were expressed in the developing meninges by double labelling these cells with antibodies to LYVE1 and LAMININ, which delineates the meninges from the parenchymal surface and surrounding tissue (Supplementary Fig. 4a, c) [10]. We conclude that murine LLEC development is not dependent on the PU.1 transcription factor, which is similar to zebrafish BLECs and unlike neighbouring macrophages [36, 66].

### Zebrafish BLECs and mouse LLECs endocytose Aβ 1-40 more efficiently than macrophages

**Fig. 5:**
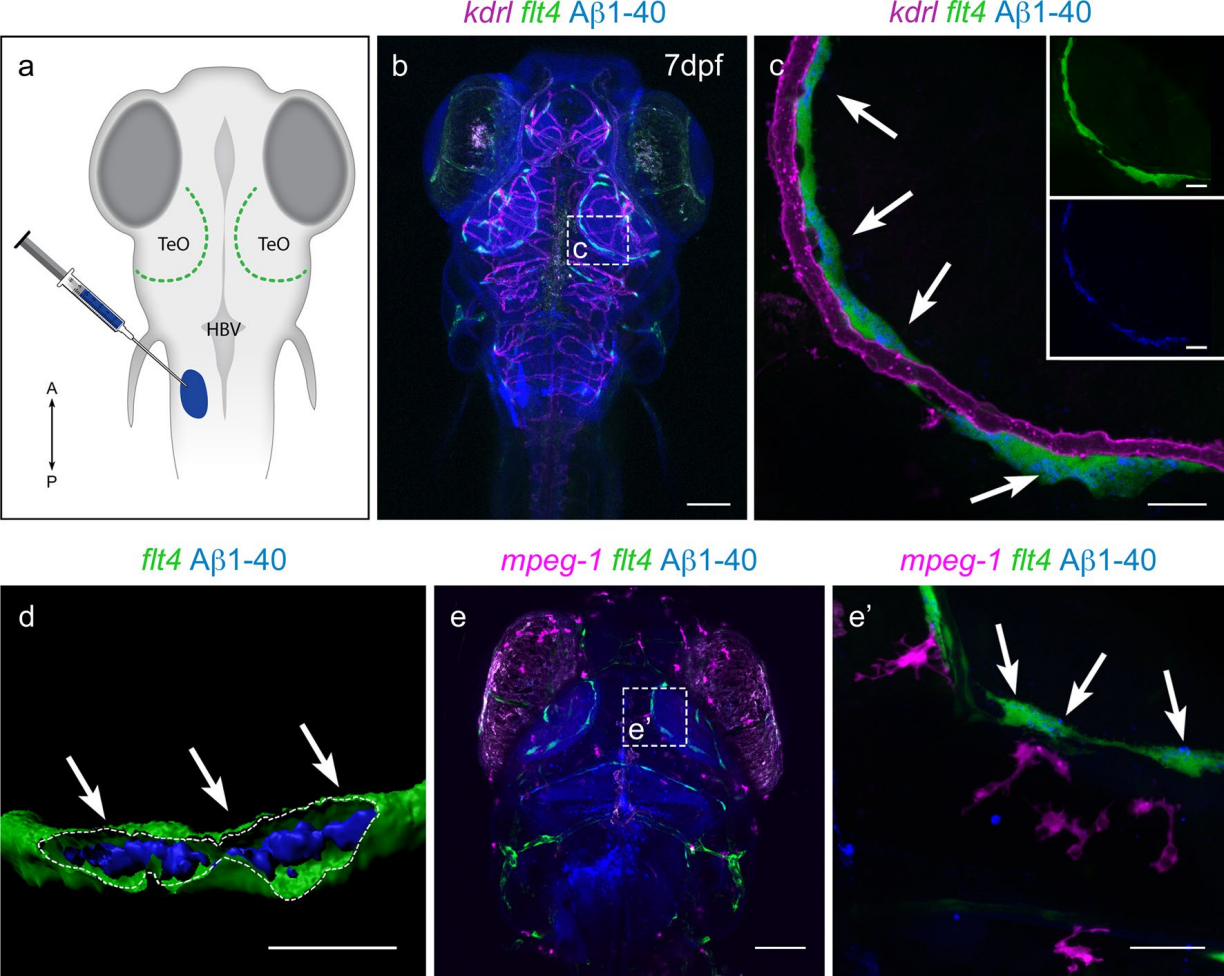
Zebrafish BLECs, but not macrophages, take up Aβ1-40. **a** Schematic showing the location of BLECs (green) relative to the Aβ1-40 injection site into a pocket of CSF near the hindbrain ventricle (HBV). TeO = Optic Tectum. *n* = 4 brains **b** Confocal projection of double transgenic *Tg(kdr*-*l:mCherry); Tg(flt4:mCitrine);casper* 7dpf larvae, which labels vasculature in red and BLECs in green, injected with Aβ1-40 (blue). Dotted box detailed in (**c**). Scale = 150 µm. *n* = 4 brains **c** After injection, Aβ1-40 (blue) accumulates within *mCitrine-*positive BLECs (green, white arrows showing co-localization) but not within *mCherry-*positive vasculature (magenta). The green (BLECs) and blue (Aβ1-40) channels are shown separately in the insets. Scale = 30 µm. *n* = 4 brains. **d** A 3-D reconstruction of the *mCitrine* positive BLEC (green) shows a cutaway (dotted line) into the interior portion of the cell to highlight the internalization of Aβ1-40 (blue with white arrows). Scale = 15 µm. *n* = 4 brains. **e** A representative confocal projection of double transgenic *Tg(mpeg1:mCherry); Tg(flt4:mCitrine);nacre*^−*/*−^ 7dpf larvae, which labels macrophages in magenta and BLECs in green, injected with Aβ1-40 (blue). The white dotted box is detailed in (**e′**) showing Aβ1-40 (blue) accumulates within *mCitrine-*positive BLECs (green) but not within *mCherry-*positive macrophages (magenta). White arrows point to co-localization of green BLECs with blue Aβ1-40. Scale = 30 µm. *n* = 3 brains

Zebrafish BLECs have the ability to internalize macromolecules up to 500 kD in size [36]. However, since not all molecules below this size limit (e.g., Evan’s Blue, molecular weight, 961 Da) can be internalized by zebrafish BLECs, we decided to examine whether disease-relevant molecules such as Amyloid β1-40 (Aβ; molecular weight, 5kD) can also be endocytosed by BLECs. We specifically chose Aβ1-40 over Aβ1-42 because Aβ1-40 is a major contributor to cerebral amyloid angiopathy (CAA, [60]) and experimentally aggregates less readily, making experiments much easier to control. Fluorescently-tagged Aβ was injected into a pocket of CSF dorsolateral to the posterior hindbrain ventricle of 7 days post fertilization (dpf) transgenic *Tg(kdr*-*l:mCherry); Tg(flt4:mCitrine);casper* zebrafish larvae, in which the vasculature is labelled with mCherry (red) and BLECs with mCitrine (green), and the subsequent distribution of Aβ was followed by live confocal imaging (Fig. 5a, c). After 30 min, Aβ accumulated in *flt4/vegfr3*-positive BLECs but not in the *kdr*-*l*-positive vasculature (Fig. 5c, d; Supplementary Video 2). To observe whether meningeal macrophages also internalized Aβ, we injected Aβ into a 7 dpf transgenic line, *Tg(mpeg1:Gal4*-*FF)*^*gl25*^*;Tg(UAS*-*E1b:nfsB.mCherry)*^*c264*^*;Tg(flt4:mCitrine);nacre*^−*/*−^, in which BLECs express mCitrine (green) and macrophages/microglia express mCherry (red). After 45 min, Aβ again accumulated in BLECs, but no Aβ was detectable in any of the nearby *mpeg1*-positive macrophages (Fig. 5e, e′). We conclude that zebrafish BLECs outperform macrophages in the acute internalization of Aβ.

**Fig. 6:**
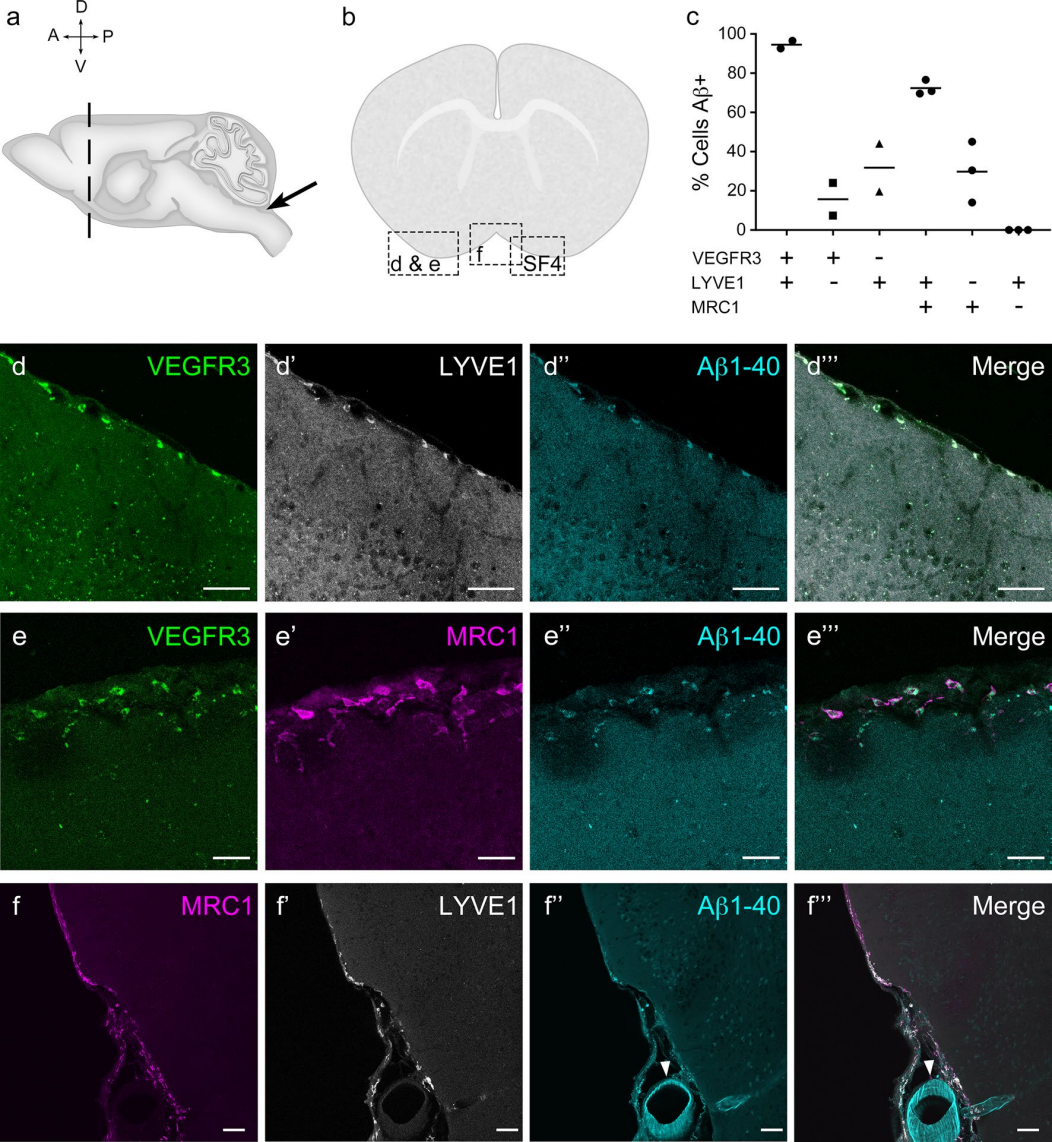
Mouse LLECs take up Aβ 1-40. **a** Schematic showing the site of dye and Aβ1-40 perfusion into the CSF via the cisterna magna (arrow) of a 2-month old mouse. The dotted line indicates the plane of section. *A* anterior, *P* posterior, *D* dorsal, *V* ventral. **b** Coronal brain section indicating the areas imaged. SF4 refers to area captured in Figure S4. **c** The percentage of each labelled cell type that internalized perfused Aβ. Cells co-expressing VEGFR3 and LYVE1 take up Aβ at a higher rate than MRC1, LYVE1 double-positive cells as well as MRC1-positive, LYVE1-negative cells (*p* ≤ 0.05, bootstrap). VEGFR3, LYVE1 counts, *n* = 2 brains (3 sections/brain). MRC1, LYVE1 counts, *n* = 3 brains (3 sections/brain). **d–d′′′** Cells of the adult mouse meninges that co-express VEGFR3 (**d**, green) and LYVE1 (**d′**, white) internalize Aβ1-40 (**d′′**, cyan). Scale = 20 µm. **e-e′′′**) Cells of the adult mouse meninges that co-express VEGFR3 (**e**, green) and MRC1 (**e′**, white) internalize Aβ1-40 (**e′′**, cyan). Scale = 40 µm. **f**–**f′′′**) Cells of the adult mouse meninges that co-express MRC1 (**f**, magenta) and LYVE1 (**f′**, white) internalize Aβ1-40 (**f′′**, cyan). The walls of a blood vessel (white arrowhead, **f′′**) also accumulate Aβ1-40. Scale = 60 µm

To test whether mouse LLECs internalize macromolecules, both 10kD cascade blue dextran and fluorescently-tagged Aβ (HiLyte-647) were slowly co-infused (1 µL/minute) into the cisterna magna of 2-month old mice (Fig. 6a, arrow) that were sacrificed after 60 min and processed for IHC. The 10kD dextran was detected within PROX1 and LYVE1 co-expressing cells (Supplementary Fig. 5), demonstrating that LLECs can internalize macromolecules. In addition to the walls of meningeal blood vessels (e.g. Fig. 6f′′), Aβ was also internalized by cells that co-expressed pairwise combinations of the BLEC associated markers MRC1, LYVE1, VEGFR3, and PROX1 (Fig. 6d–f′′′ and Supplementary Fig. 5). Although technical constraints precluded the simultaneous imaging of fluorescently-tagged Aβ and three LLEC markers, the high rate of LLEC marker co-localization (often > 90% e.g. see Fig. 1 and Supplementary Fig. 1) in the leptomeninges means the uptake rates observed in these pairwise combinations is a strong indication that LLECs expressing at least four markers are internalizing Aβ.

Since Aβ is preferentially internalized by BLECs and not by macrophages in zebrafish, we quantified the rates of Aβ internalization by these populations. On average, 95% of mouse LLECs, which express both VEGFR3 and LYVE1, had internalized Aβ after 60 min (Fig. 6c). In contrast, 72% of MRC1, LYVE1 double positive cells, which identifies both putative BLECs and macrophages, internalized Aβ, a significantly lower rate compared to LLECs (*p* < 0.05, bootstrap). The rates of Aβ internalization were even lower for cells expressing only MRC1, with only 30% of these cells internalizing Aβ (Fig. 6c). Together with the zebrafish data, these results suggest that a greater capacity to internalize Aβ is a hallmark feature of BLECs and LLECs that functionally distinguishes them from meningeal macrophages.

### BLEC/LLEC markers are co-expressed in cells of the human meninges

**Fig. 7:**
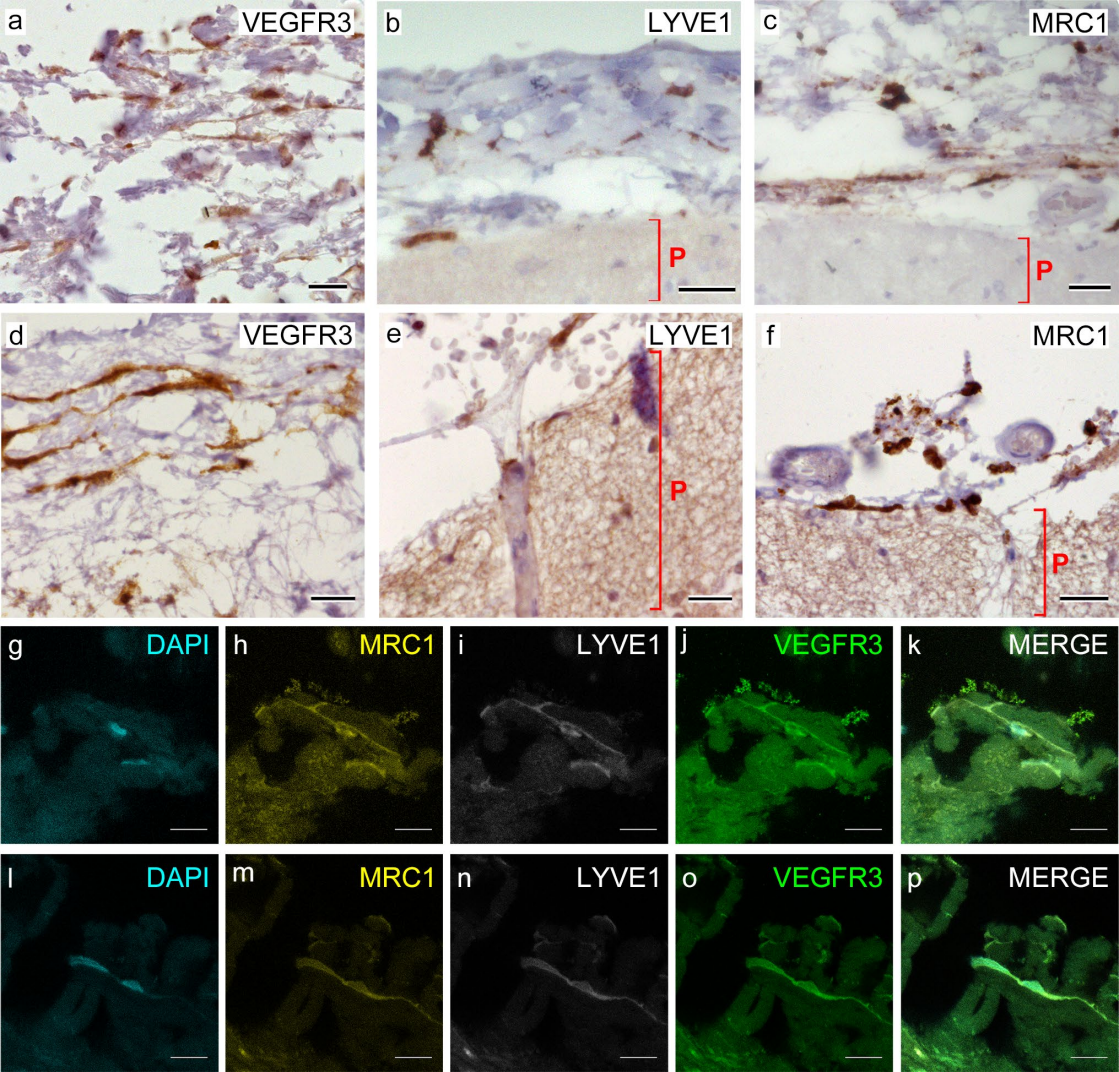
Cells of human meninges co-express LLEC markers.** a**–**c** DAB-IHC with single antibodies detects VEGFR3 (**a**), LYVE1 (**b**), and MRC1 (**c**) in the meninges of human post mortem brain showing no signs of neuropathology. These images are taken from a 38 year old male (sample P17/07, Table 1), and confirmed in n = 2 additional samples. *P* parenchyma. Scale = 150 µm (**a**); 40 µm (**b**); and 20 µm (**c**). **d**–**f** DAB-IHC with single antibodies detects VEGFR3 (**b**), LYVE1 (**c**), and MRC1 (**d**) in elderly human meninges (age: 89–92) with evidence of neuropathology and confirmed in *n* = 3 brains (Table 1). P, parenchyma. Scale = 20 µm. **g–p** IHC with fluorescent antibodies detects human meningeal cells that co-express MRC1 (**h**, **m**, yellow), LYVE1 (**i**, **n**, white), and VEGFR3 (**j**, **o**, green). Nuclei/RNA are labelled with DAPI (**g**, **l**, blue) and images are merged in (**k**, **p**). Scale = 10 µm

To determine whether LLECs are also present in human meningeal tissue, we performed immunostaining with antibodies against LLEC proteins on sections from post-mortem human brains that lacked obvious neuropathology (Table 1). As in zebrafish and in mice, VEGFR3, LYVE1 and MRC1 were all found to be expressed within a heterogeneous population of cells within the pia and subarachnoid space of the leptomeninges of these samples (Fig. 7a–c). We then examined post-mortem meninges obtained from elderly human samples (aged 88–92) showing various degrees of neuropathology (Table 1) and again found VEGFR3, LYVE1, and MRC1 expression (Fig. 7d–f, and Supplementary Fig. 6). VEGFR3 was also strongly expressed in the lining of leptomeningeal vascular walls (Supplementary Fig. 7e, black arrows), in a tight layer of interdigitated cells comprising the arachnoid barrier (Supplementary Fig. 7d, orange arrows) [63, 70], and in clusters of cells corresponding to the arachnoid trabeculae (Supplementary Fig. 7b), including cells with pale nuclei with small prominent nucleoli typical of leptomeningeal and meningioma cells (Supplementary Fig. 7c) [12, 62]. This helps to identify these cells as phenotypically leptomeningeal cells. These observations raise the possibility that human leptomeninges contain LLECs, although the expression of some markers such as VEGFR3 is more widespread in other meningeal tissue than observed in fish and rodents.

To determine if the cells in the pia and subarachnoid space that express lymphatic markers represent LLECs, we performed triple-labelling fluorescent IHC on post-mortem human brain tissue for MRC1, LYVE1, and VEGFR3. We found several examples of these three LLEC markers co-expressed in the same leptomeningeal cells (Fig. 7g–p and Supplementary Video 3). To emphasize the spatial locations of LLECs relative to vessels and each other and to more clearly make out their morphological features, we used the Imaris imaging software to make 3-D reconstructions from the serial confocal images of the leptomeningeal human post-mortem tissue (Supplemental Fig. 5e–h). Consistent with LLECs in zebrafish and mouse, a subset of triple-positive cells were closely associated with vessels within the leptomeningeal layer, while their cell bodies remained outside of the vascular basement membrane. Morphologically, these cells were similar to zebrafish and mouse BLECs, with long, thin processes near blood vessels. Several of these processes formed unusual loop-like structures of unknown functional significance that were also a common morphological feature observed in zebrafish BLECs (Supplementary Fig. 8).

Taken together, the co-expression of three lymphatic markers in subarachnoid cells with a distinctive morphological character supports the existence of LLECs in human leptomeninges.

## Discussion

BLECs were originally described as a non-lumenized endocytic cell type of lymphatic developmental origin and molecular expression that resides in the zebrafish meninges [11, 36, 66]. Here, we described LLECs, cells with similar molecular, ultrastructural, developmental, and functional characteristics as BLECs in both mouse and human leptomeninges. The location and functional properties of LLECs indicate potential role(s) for these cells in the immunological organization of this meningeal compartment and in the regulation of CSF protein homeostasis.

### LLECs are distinct from other known cell types

We provide evidence that zebrafish BLECs have an equivalent cell type in both mouse and humans that we have named LLECs. Do LLECs represent a unique cell type? Although cells expressing the lymphatic markers of BLECs have not to our knowledge been previously described in the leptomeninges, there are three other meningeal cells types that share at least a few LLEC features, including Mato Cells/FGPs, meningeal macrophages, and telocytes [8, 16, 40]. We consider whether LLECs are equivalent to, or a subpopulation of, each of these cell types in turn.

#### Mato cells/FGPs

One of the initial characterizations of zebrafish BLECs suggested these were the fish homolog of Mato Cells/FGPs mainly based on the presence of large, auto-fluorescent inclusions [66]. However, we have been unable to identify auto-fluorescent inclusions in either zebrafish or mouse, suggesting that auto-fluorescence is not a consistent feature of BLECs, perhaps reflecting dietary differences affecting lipid content within such inclusions [42]. Additionally, ultrastructural analysis of Mato Cells/FGPs has described the inclusions as more heterogeneous in size and shape, with many taking on a foamy appearance. In contrast, immuno-EM labelled BLECs in both fish and LLECs in mice contain mostly large, round, uniformly electron-dense inclusions (Fig. 2b), with some neighbouring cells more closely resembling FGPs (Fig. 2e–g). Finally, FGPs are described as positioned between the smooth muscle cells and the glia limitans [42], whereas BLECs in zebrafish and LLECs in mice remain external to vascular basement membranes (Fig. 2a, b) [36]. Although we cannot rule out the possibility that LLECs are a subpopulation of Mato Cells/FGPs, perhaps one that has dynamically changed in response to unknown cues, our data indicate that Mato Cells and LLECs are distinct cell types in the vertebrate meninges.

#### Meningeal macrophages (mMΦs)

While LLECs and mMΦs express some of the same genes, including MRC1, the lymphatic and LLEC markers PROX1 and VEGFR3 have not previously been described in mMΦs (Fig. 1, 7, Supplementary Fig. 1&5). Thus, although they share some morphological and phagocytic characteristics, LLECs and mMφs likely play non-overlapping roles in meningeal surveillance and maintenance. Consistent with this, in both zebrafish and mouse, BLECs/LLECs more efficiently internalized Aβ than neighboring mMΦs (Fig. 5, 6, Supplementary Fig. 5).

We also found that LLECs and mMφs do not share a developmental origin. In zebrafish, both lineage tracing and mutagenesis of signalling genes involved in the specification of lymphatic endothelium established a lymphatic origin for BLECs [11, 36, 66]. Zebrafish BLECs also developed normally in the absence of the pU.1 transcription factor, which is essential for the development of *mpeg1*-positive macrophages. In mice, MRC1^+^ mMΦs are derived from the yolk sac or liver in a PU.1-dependent manner and begin colonizing the leptomeninges from E9.5 [18]. In contrast to mMΦs but similar to zebrafish BLECs, we detected LYVE1, MRC1, and PROX1 triple-positive cells at E15.5 in PU.1 knock-out mice (Fig. 4), demonstrating that mouse LLECs develop independent of this essential mMΦs transcription factor.

Together, the molecular, developmental, and functional data indicate that LLECs and mMΦs are distinct classes of meningeal cells.

#### Telocytes

Telocytes are a recently discovered stromal interstitial cell type found in many organs including the heart [48, 52], intestines [64] and uterus [47]. Telocytes are characterized by their small cell bodies, unusually long and fine processes, and their close association with vasculature. Telocytes are often mistaken as other stromal cell types such as fibroblasts, especially in the meninges and choroid plexus [9, 46]. However, telocytes are not noted for large intracellular inclusions, phagocytic/endocytic properties, or expression of lymphatic-associated markers as seen in LLECs [8, 16]. In addition, the processes of LLECs are not as long and fine as in telocytes, which can be hundreds of µm long [15] and as thin as 0.2 μm [65]. LLECs and telocytes are therefore likely to be distinct classes of cells.

### Potential roles for BLECs in the surveillance and clearance of CSF solutes

The ability of BLECs to readily endocytose macromolecules up to 500 kD in an Mrc1-dependent manner [36] and for mammalian LLECs to internalize dyes of at least 10 kD suggests that they participate in the surveillance and clearance of CSF solutes. Mrc1 is a pattern recognition receptor that is involved in the identification, binding, and endocytosis of pathogens, including bacteria involved in tuberculosis (Mycobacterium tuberculosis) and meningitis (Streptococcus pneumoniae) and fungi such as *Candida albicans* [6, 17, 49, 54, 56, 57]. In other cellular contexts, MRC1 can be exploited by pathogens. For example, viruses such as Influenza type A can enter and infect MΦs via MRC1 [50], and Mycobacterium tuberculosis can co-opt lymphatic endothelial cells in lymph nodes to serve as a replicative niche following MRC-dependent endocytosis [35]. Whether LLECs also participate in the uptake of pathogens or can serve as a reservoir for infection remains to be determined. MRC1 also participates in immune organization, for example by trafficking lymphocytes along lymphatic endothelium to draining lymph nodes, a process also exploited by tumours to metastasize to nodes [41]. Whether the densely interconnected network of LLECs that reside in the meninges perform a similar immune organizational function, for example by providing signalling cues or sites of attachment for lymphocytes [53], is an interesting question for future investigation.

While BLECs and LLECs can internalize a diversity of macromolecules, the selective ability of BLECs/LLECs to internalize Aβ more readily than meningeal macrophages suggests that these cells may especially participate in the clearance of Aβ, which accumulates within the wall of leptomeningeal arteries in CAA [68]. Because CAA may commence with Aβ accumulation in leptomeningeal vessels during aging as well as in Alzheimer’s Disease patients [19, 55], and cases of CAA solely within the leptomeninges have been reported [58], LLECs are well positioned to participate in the progression of this disease. Interestingly, chemical ablation of MRC1-expressing perivascular macrophages (pvMφ) using mannosylated clodronate containing liposomes exacerbates the development of CAA in a mouse model [20]. Since MRC1-expressing LLECs would likely also be eliminated by this methodology, this result should be revisited to examine whether LLECs participate in playing a protective role against Aβ-related disease. If so, whether LLECs have been lost or have diminished function in patients with CAA should also be examined.

More generally, the discovery of LLECs expands the diversity of “brain” lymphatics beyond the lymphatic vessels of the dura mater to also include cells within the leptomeninges. Although the role of dural brain lymphatics in the clearance of the brain and CSF remains controversial, there are several possibilities by which LLECs may interact with dural lymphatics to participate in the drainage and macromolecular clearance of the CSF that warrant further exploration. First, although under normal conditions the LLECs are not lumenized like the dural lymphatics, zebrafish BLECs can transiently form tubes that penetrate the brain parenchyma upon brain vascular injury [13]. Whether mammalian LLECs can also form tubes in certain disease or injury contexts to directly participate in lymphatic drainage of the brain should be examined in future studies. Second, the loosely interconnected meshwork of BLECs/LLECs could physically interact with dural lymphatics to participate in solute removal. For example, fluorescent tracer imaging experiments have revealed lymphatic articulations from the dura into the subarachnoid space near tracer “hot spots” at several points along the skull [37], and a recent paper has characterized a set of dural lymphatic vessels in the base of the mouse skull that have valves and other structures of classical lymphatics, lie close to the subarachnoid space, and reside in a thinner part of the dura mater [1]. Whether the distribution of LLECs within the leptomeninges shows a similar intracranial variation is unknown, but holocranohistochemistry to preserve anatomical architecture [60] could facilitate mapping the positions of LLECs relative to dural lymphatic articulations and other important structures of the meninges.

The ability of LLECs to participate CSF macromolecular clearance (at least under experimental conditions) also makes them potentially interesting targets for clinical applications. For example, enhancing dural lymphatic development using soluble VEGF improved macromolecule drainage and cognitive impairment in an AD mouse model [44]. How the VEGFR3-expressing LLECs respond to VEGF signalling is not known, but LLECs may also have been affected alongside the dural lymphatics when manipulated with VEGFR signalling [1, 44]. Whether LLEC number or endocytic function can be enhanced, and whether this has a protective effect in animal models of CAA, AD, or other pathological states would not only demonstrate a critical role for LLECs in disease progression but would also highlight LLECs as a promising target for therapeutic intervention.

## Electronic supplementary material

Below is the link to the electronic supplementary material.

Supplementary material 1 (PDF 1794 kb)

Supplementary material 2 (MOV 7689 kb)

Supplementary material 3 (MP4 859 kb)

Supplementary material 4 (MOV 14216 kb)
