# Supplementary material for: Structural and functional conservation of non-lumenized lymphatic endothelial cells in the mammalian leptomeninges
Source: Acta Neuropathol. 2019 Nov 6;139(2):383–401. doi: 10.1007/s00401-019-02091-z (PMC6989586; doi:10.1007/s00401-019-02091-z)
Supplement: Supplementary file 1 — Supplementary material 1 (PDF 1794 kb) [file 401_2019_2091_MOESM1_ESM.pdf]

Supplementary Figures and Videos, Online Resource.

Shibata-Germanos et al., “Structural and functional conservation of non-lumenized lymphatic endothelial cells in the mammalian leptomeninges”.

Contents:

8 Supplementary Figures and Legends

3 Supplementary Video Descriptions

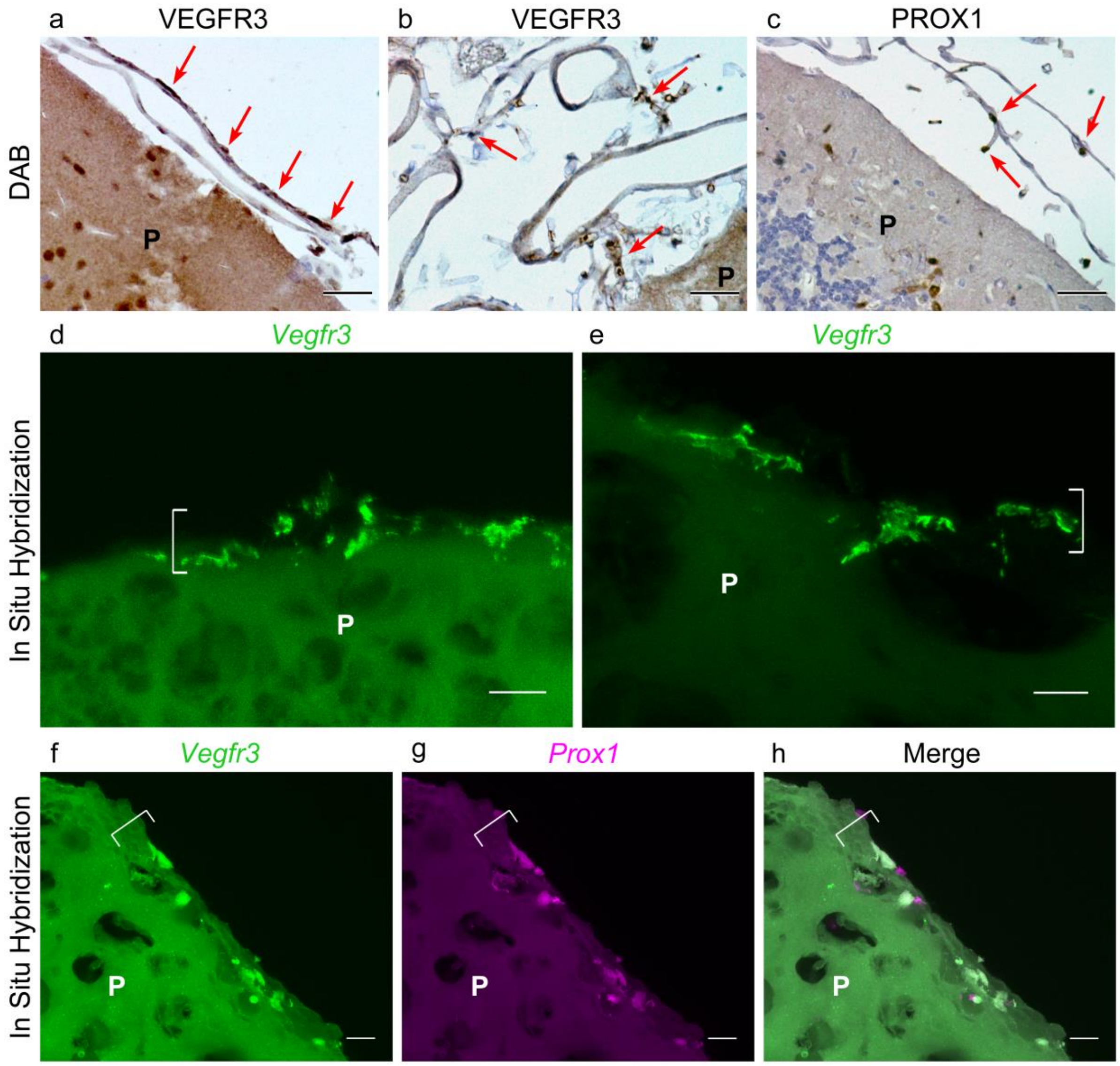

Supplementary Fig. 1. Cells of Murine Meninges co-express *Vegfr3* and *Prox1* mRNA.

a-c) DAB-IHC on paraffin embedded coronal 6-month old mouse brain sections using alternative antibodies to **Figure 1** and **6** detects VEGFR3-positive (**a-b**) and PROX1-positive (**c**) cells (red arrows) throughout the leptomeninges. P, Parenchyma; Scale=10 µm; n=2. **d-e**) Fluorescent in situ hybridization against *Vegfr3* mRNA labels cells in the 2-month-old mouse meninges (white bracket). Scale=20 µm; n=2. **f-h**) Double fluorescent in situ hybridization detects co-localization of *Vegfr3* (**f**, green) and *Prox1* (**g**, magenta) mRNA within the 2-month old mouse meninges (white bracket). Merged in (**h**). Scale=20 µm; n=2.

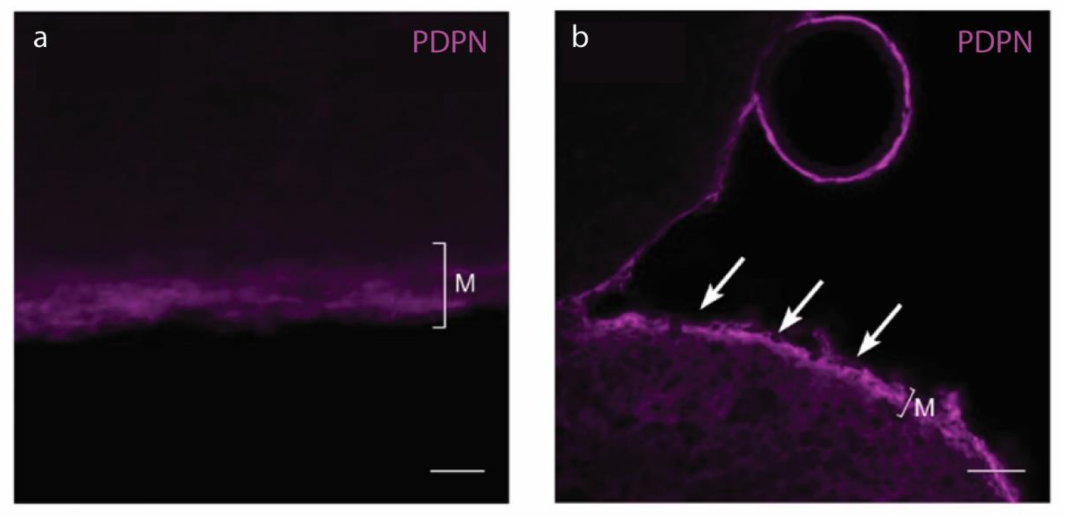

Supplementary Fig. 2. PDPN is broadly expressed in the mouse meninges.

a-b) An anti-PDPN antibody ubiquitously labels the pial layer (white arrows) in 2 month old mouse sections. M=meninges. Scale=10 µm (a), 50 µm (b). n=2

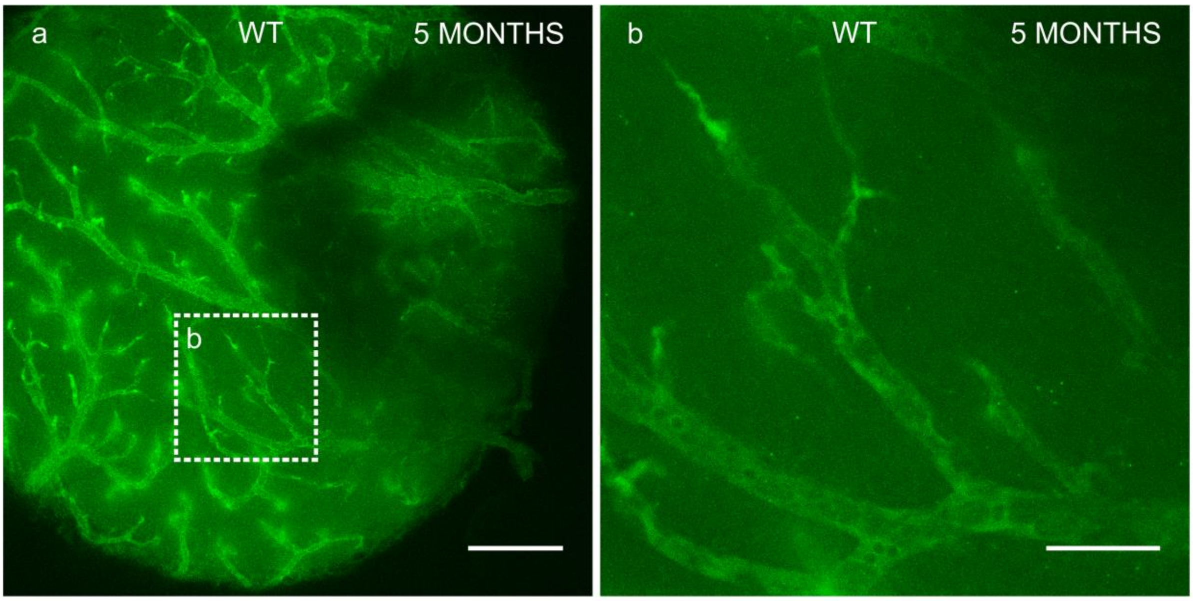

Supplementary Fig. 3. Zebrafish BLECs Do Not Have Auto Fluorescent Inclusions.

a) Confocal projection from applying a 500-588nm wavelength bandpass with the 488nm laser to the dorsal surface of a whole mount five-month-old wildtype zebrafish optic tectum. The vasculature shows strong autofluorescence in green. The white dotted box denotes the area imaged in panel (**b**). Scale=100µm; n=3.

b) A higher magnification reveals auto-fluorescent vasculature without notable fluorescent inclusions in the space around the vessels where BLECs reside in the dorsal surface of the adult zebrafish optic tectum. Scale=30µm.

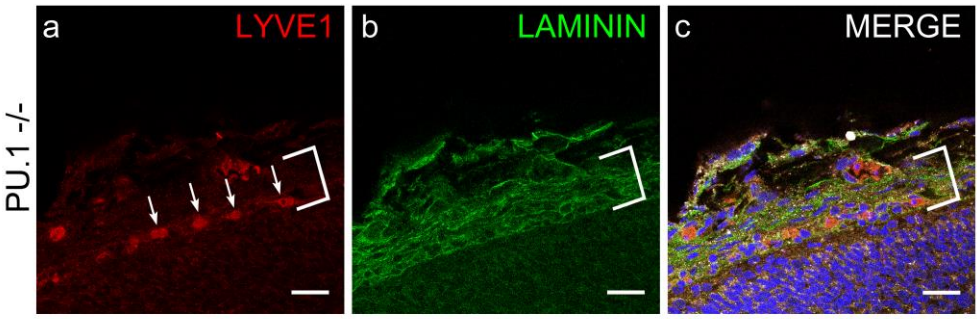

Supplementary Fig. 4. PU.1 knockout mice brain sections reveal LYVE1+ cells within the LAMININ rich meninges.

a-c) IHC on E15.5 PU.1 knockout mice reveals LYVE1-positive cells (**a**, red with white arrows) within the LAMININ positive meningeal layers (**b**, green). DAPI-labelled nuclei are blue in (**c**). The white brackets delimits the meninges. Scale=30µm; n=3.

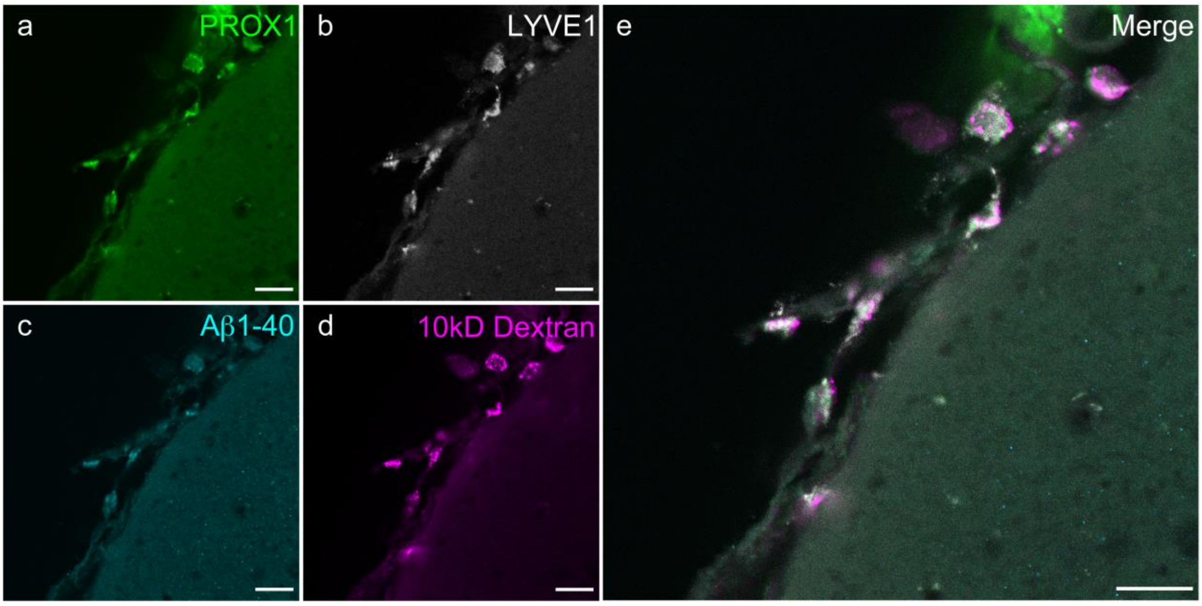

Supplementary Fig. 5. LLECs co-expressing PROX1 and LYVE1 internalize Aβ1-40 and 10kD Dextran.

a-e) Following the co-perfusion of Aβ1-40 and 10kD Dextran into the cisterna magna of a two month old mouse, IHC reveals mouse meningeal cells that co-express PROX1 (**a**, green) and LYVE1 (**b**, white) that have internalized both Aβ1-40 (**c**, cyan) and 10kD Dextran (**d**). Merged in (**e**). Scale=20µm; n=3.

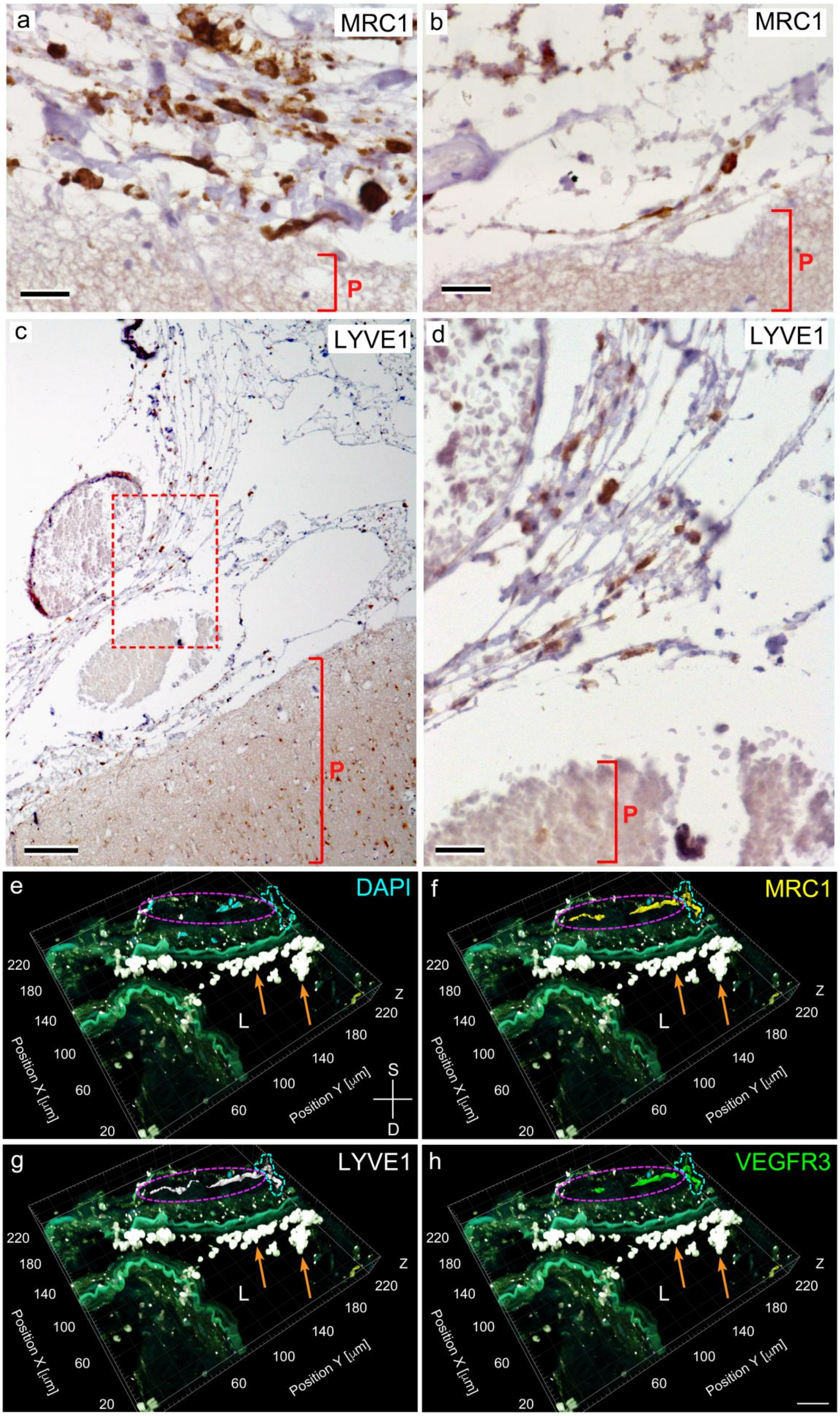
a
MRC1
P
b
MRC1
P
c
LYVE1
P
d
LYVE1
P
e
DAPI
L
Position X [μm]
Position Y [μm]
Z
S
D
f
MRC1
L
Position X [μm]
Position Y [μm]
Z
g
LYVE1
L
Position X [μm]
Position Y [μm]
Z
h
VEGFR3
L
Position X [μm]
Position Y [μm]
Z

Supplementary Fig. 6. LLEC associated markers are expressed in elderly human leptomeninges.

a-b) Additional examples of MRC1 positive cells detected in coronal sections of elderly human leptomeninges with DAB-IHC using antibodies against MRC1. Related to Figure 7. P, parenchyma. Scale= 20µm; n=3

c-d) Additional examples of LYVE1 positive cells detected in coronal sections of human leptomeninges by DAB-IHC. Related to Figure 7. The red dotted box in **c**) is shown at higher magnification in **d**). Scale= 20 µm **(c),** =50 µm **(d)**; n=3.

e-h) 3-D reconstructions from a series of confocal stacks taken from a post-mortem human brain sample show the relative position of the triple-labelled (MRC1+;LYVE1+;VEGFR3+) examples of LLECs in Figure 7 to each other and their close association with blood vessels (green auto-fluorescence). The magenta dotted circle highlights the cells shown in Figure 7 **g-j**) and the cyan dotted circle indicates the cell from Figure 7 **k-m**) Note the long cellular processes of the LLECs. Arrows indicate blood cells. L, lumen. For the compass in (**e**), S=superficial, D=deep.

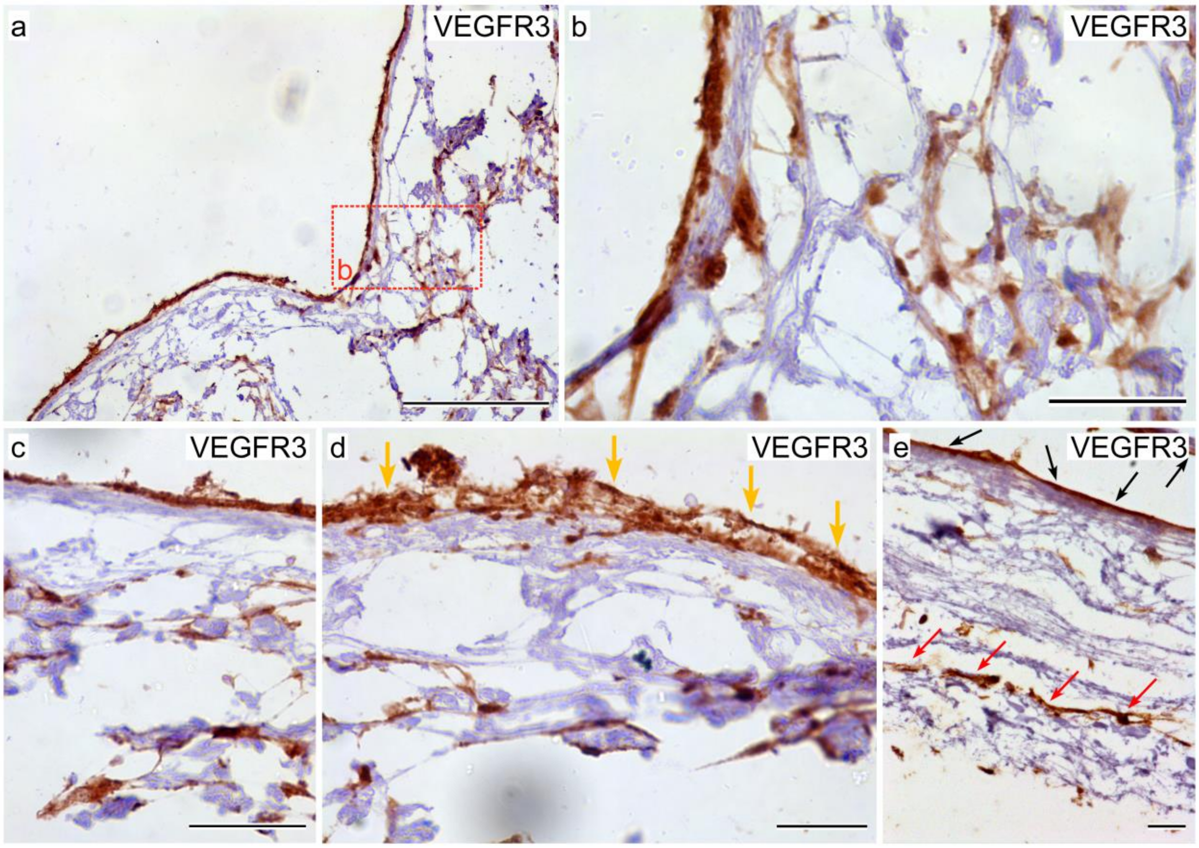

Supplementary Fig. 7. VEGFR3 expression in elderly human leptomeninges.

a-b) An additional example of VEGFR3 expression in post mortem human meningeal tissue detected by DAB-IHC. The red box in **(a)** is shown in higher magnification in (**b**) to highlight VEGFR3 positive cells in the subarachnoid space. Related to Figure 7.

c) As detected by DAB-IHC, VEGFR3 is expressed in cells with pale nuclei with small prominent nucleoli typical of leptomeningeal cells and meningioma cells. This helps to identify these cells as phenotypically leptomeningeal cells.

d) αVEGFR3 antibodies strongly label the arachnoid barrier (orange arrows), as detected by IHC.

e) As revealed by DAB-IHC, human leptomeningeal vessel walls are lined with VEGFR3 expression (black arrows) and VEGFR3 positive leptomeningeal cells can be found in deeper tissue (red arrows). Scale= 50 µm **(a)**, =10 µm **(b-e)**; n=3.

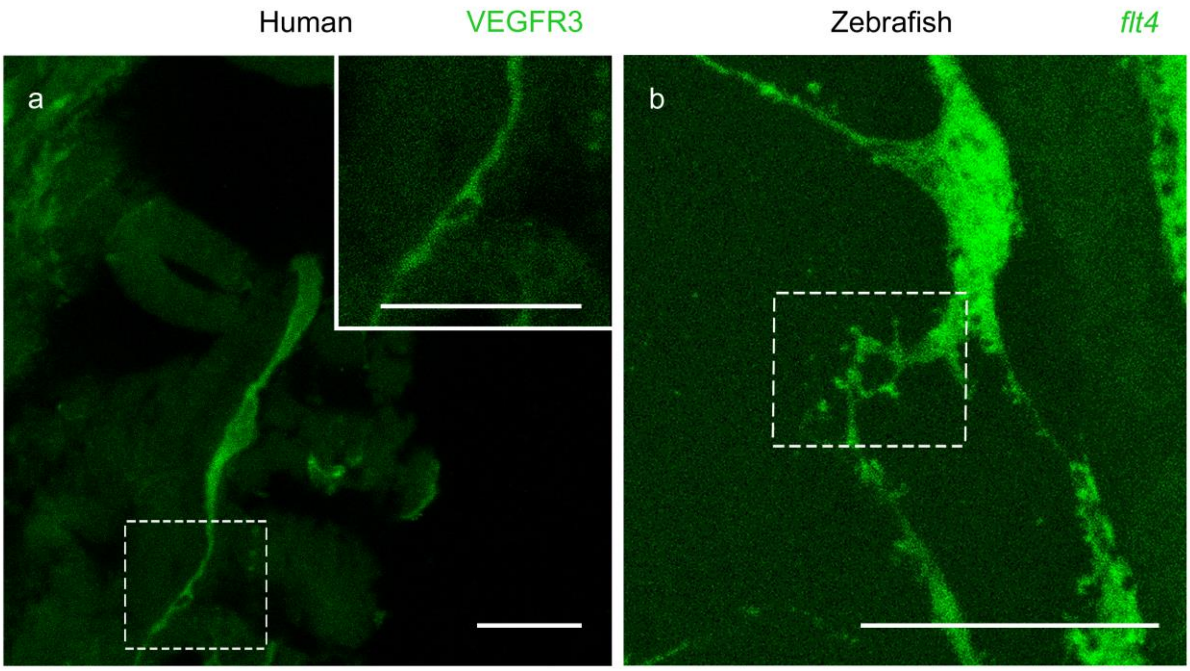

Supplementary Fig. 8. Morphological loops in the cellular processes of human and zebrafish VEGFR3 positive meningeal cells.

a) IHC with fluorescent antibodies against VEGFR3 (green) detects morphological features of human LLECs. The VEGFR3-expressing leptomeningeal cells often form looping processes (white dotted box area of inset) that are also found in zebrafish BLECs (see b). Scale= 10µm.

b) In adult (4 month old) transgenic *Tg(flt4:mCitrine)* zebrafish, *flt4 (vegfr3)* positive BLECs (green) also form looping processes (white dotted box is area of interest), as seen in this whole mounted confocal image . Scale=30µm.

Supplementary VIDEO 1. Mouse LLECs do not have auto fluorescent inclusions.

A confocal z-stack of the cortical surface of a five-month-old wildtype mouse brain while applying a 500-588nm bandpass with the 488nm laser (dorsal view). Related to Supplemental Figure 3. n=3.

Supplementary VIDEO 2. Zebrafish BLECs can internalize Aβ 1-40.

A 3-D Imaris rendered image of a *Tg(kdr-l:mCherry); Tg(flt4:mCitrine)* double transgenic showing zebrafish BLECs (Green) adjacent to the blood vasculature (purple) on the meningeal surface of the optic tectum. Aβ1-40 (blue) is detected within the green BLECs. Related to Figure 5.

Supplementary VIDEO 3. Human meningeal tissue reveals cells expressing LLEC markers near a leptomeningeal vessel.

Z-stack of post-mortem human meningeal tissue triple labelled by fluorescent IHC with antibodies against MRC1, LYVE1, and VEGFR3. Triple-labelled LLECs are visible near the leptomeningeal vasculature. Related to Figure 7.
